# The *in vivo* functional significance of PUF hub partnerships in *C. elegans* germline stem cells

**DOI:** 10.1242/dev.201705

**Published:** 2023-05-09

**Authors:** Ahlan S. Ferdous, Stephany J. Costa Dos Santos, Charlotte R. Kanzler, Heaji Shin, Brian H. Carrick, Sarah L. Crittenden, Marvin Wickens, Judith Kimble

**Affiliations:** Department of Biochemistry, University of Wisconsin-Madison, Madison, WI 53706, USA

**Keywords:** Intrinsically disordered proteins, RNA repression, CNOT complex, Network hub

## Abstract

PUF RNA-binding proteins are conserved stem cell regulators. Four PUF proteins govern self-renewal of *Caenorhabditis elegans* germline stem cells together with two intrinsically disordered proteins, LST-1 and SYGL-1. Based on yeast two-hybrid results, we previously proposed a composite self-renewal hub in the stem cell regulatory network, with eight PUF partnerships and extensive redundancy. Here, we investigate LST-1–PUF and SYGL-1–PUF partnerships and their molecular activities in their natural context – nematode stem cells. We confirm LST-1–PUF partnerships and their specificity to self-renewal PUFs by co-immunoprecipitation and show that an LST-1(A^m^B^m^) mutant defective for PUF-interacting motifs does not complex with PUFs in nematodes. LST-1(A^m^B^m^) is used to explore the *in vivo* functional significance of the LST-1–PUF partnership. Tethered LST-1 requires this partnership to repress expression of a reporter RNA, and LST-1 requires the partnership to co-immunoprecipitate with NTL-1/Not1 of the CCR4-NOT complex. We suggest that the partnership provides multiple molecular interactions that work together to form an effector complex on PUF target RNAs *in vivo*. Comparison of LST-1–PUF and Nanos–Pumilio reveals fundamental molecular differences, making LST-1–PUF a distinct paradigm for PUF partnerships.

## Teaser

**Summary:** Partnerships between PUF RNA-binding proteins and intrinsically disordered proteins are essential for stem cell maintenance and RNA repression in *C. elegans*.

## INTRODUCTION

RNA-binding proteins are central to gene regulation and a wide range of biological phenomena and human diseases (Gebauer et al., 2021; Gong et al., 2022; Matia-González et al., 2015). Often, they work within regulatory complexes that modulate their activity. Most relevant to this work, PUF RNA-binding proteins (PUF for Pumilio and FBF) are broadly conserved regulators of gene expression. From yeast to humans, PUF proteins bind mRNAs with exquisite sequence specificity, and repress RNA stability or translation (Goldstrohm et al., 2018; Miller and Olivas, 2011; Wickens et al., 2002; Zamore et al., 1997). Moreover, PUF proteins have conserved biological roles in stem cells and neurobiology, with recently discovered links to human disease (Gennarino et al., 2018; Gong et al., 2022; Naudin et al., 2017; Rajasekaran et al., 2022). Great progress has been made with respect to understanding the molecular function of PUF proteins themselves, but PUFs interact with numerous other proteins (Campbell et al., 2012a,b; Friend et al., 2012; Ginter-Matuszewska et al., 2011; Jaruzelska et al., 2003; Moore et al., 2003; Wu et al., 2013). Partnership with Nanos, for example, enhances the binding affinity of PUF proteins to RNA and refines its recognition sequence (Weidmann et al., 2016). The functions of other partnerships, however, are poorly characterized and represent the next frontier in understanding how PUF proteins control gene expression.

**Fig. 1.**
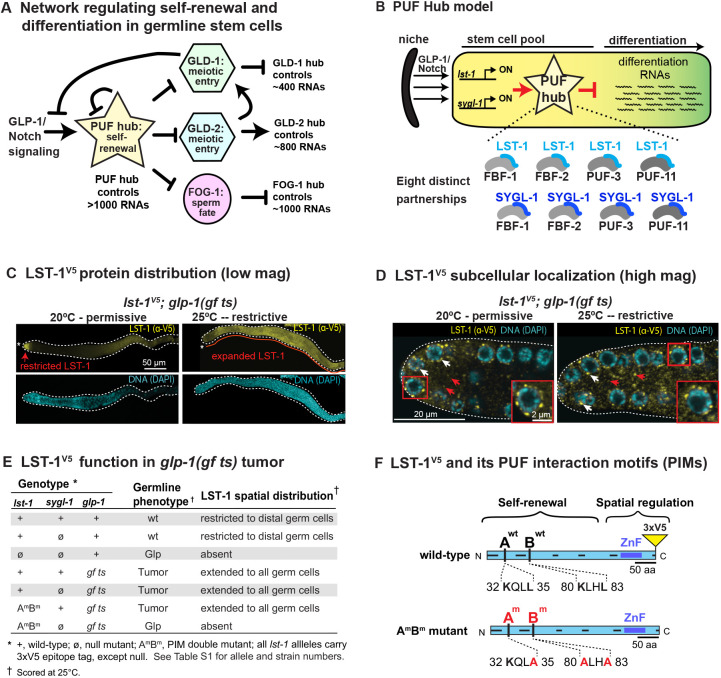
**PUF partnerships in the PUF hub and their biochemical analysis in nematodes. (**A) GSC regulatory network. This simplified diagram depicts major regulatory hubs and how they relate to each other via repression (blunted line) or activation (arrow). The hubs control many hundreds to >1000 RNAs and thus promote GSC self-renewal (PUF hub) or differentiation (GLD-1, GLD-2 and FOG-1 hubs). See Kershner et al. (2013) and Hubbard and Schedl (2019) for more complete views. (B) PUF hub model. LST-1 and SYGL-1 are central to a composite regulatory hub: each is proposed to partner with any of four PUF proteins (FBF-1, FBF-2, PUF-3 and PUF-11; gray) and to repress differentiation RNAs for maintenance of the GSC pool. GLP-1/Notch signaling activates *lst-1* and *sygl-1* transcription (black arrows) at the distal end of the gonad, which restricts LST-1 and SYGL-1 expression to the GSCs*.* (C) LST-1^V5^ distribution expands in *glp-1(gf ts)* mutants. Representative confocal *z*-projections of extruded gonads stained with a V5 antibody to detect LST-1^V5^ (yellow) and with DAPI (cyan) for DNA. Red arrow marks spatially restricted LST-1^V5^ at permissive temperature, as in wild type (Haupt et al., 2019; Shin et al., 2017); red line marks expanded LST-1^V5^ in a germline tumor, formed at restrictive temperature. Asterisk indicates distal end of the gonad, and dotted line marks its boundary. (D) Subcellular distribution of LST-1^V5^ in *glp-1(gf ts)* germline. Representative images of single confocal *z*-slices from the middle plane of distal region of extruded gonads stained with V5 antibody to detect tagged LST-1^V5^ (yellow). LST-1^V5^ is detected in both perinuclear puncta (white arrows) and the cytoplasm (red arrows). Inset shows higher magnification of the boxed area. (E) LST-1^V5^ retains stem cell regulatory function when assayed in the absence of SYGL-1, both in a normal germline (row 2) and when expanded in *glp-1 (gf ts)* germline tumors (row 4), but not when it lacks its PUF-interacting motifs (row 6). (F) LST-1 protein architecture. LST-1 possesses an N-terminal ‘self-renewal’ domain composed of multiple IDRs (black lines along protein axis) and a C-terminal ‘spatial regulation’ domain with additional IDRs and a zinc finger (ZnF; ultramarine blue). Within the self-renewal region are two PUF interaction motifs (A and B), shown with wild-type (top) and mutant (bottom) sequences.

A protein–RNA regulatory network drives self-renewal and differentiation of germline stem cells (GSCs) in the nematode *Caenorhabditis elegans* (Kershner et al., 2013). Notch signaling from the stem cell niche activates the network, and several network hubs regulate many RNAs, each other and Notch signaling (Fig. 1A) (Austin and Kimble, 1987; Kershner et al., 2014). The ‘PUF hub’ model has been proposed to describe a composite node in the GSC regulatory network composed of eight distinct PUF–partner complexes (Fig. 1B) (Haupt et al., 2020; Shin et al., 2017). Molecular evidence for eight PUF partnerships was based on assays in yeast or *in vitro*, all done with incomplete protein fragments (Haupt et al., 2020; Qiu et al., 2019, 2022; Shin et al., 2017). Although those experiments were powerful, a deep understanding of how PUF proteins regulate RNAs in stem cells demands testing their molecular activities in the cells where they normally act. In this work, we investigate PUF hub partnerships in their natural context *– C. elegans* GSCs – and do so for full-length proteins with validated biological functions. As an introduction to this complex mesh of regulators, we first describe the key PUF proteins, then the partners and finally their partnerships.

Four PUF proteins belong to the self-renewal hub (Crittenden et al., 2002; Haupt et al., 2020) (Fig. 1A). FBF-1 and FBF-2 are nearly identical to each other and play the more prominent role; PUF-3 and PUF-11 also have sequences similar to each other but play a more minor role. Like other PUF proteins, the four self-renewal PUFs bind to sequence elements in the 3′UTR of their target mRNAs (Hubstenberger et al., 2012; Koh et al., 2009; Zhang et al., 1997) and are best known for repression of differentiation RNAs in the GSC pool (Crittenden et al., 2002; Merritt et al., 2008). The four PUFs are variably redundant with each other: no major GSC defect occurs in any of the single mutants (*fbf-1*, *fbf-2*, *puf-3* or *puf-11*) or in the *puf-3 puf-11* double mutant. However, all GSCs are lost to differentiation at the last larval stage in *fbf-1 fbf-2* double mutants, and in early larvae of *fbf-1 fbf-2; puf-3 puf-11* quadruple mutants. Thus, these four PUF proteins are responsible for GSC self-renewal throughout development.

Two PUF partners, LST-1 and SYGL-1, also belong to the hub (Haupt et al., 2020; Kershner et al., 2014; Shin et al., 2017) (Fig. 1A). Both proteins are composed largely of regions of low complexity, which are predicted to be intrinsically disordered (IDRs). The two proteins bear no sequence similarity, but they are functionally redundant: no major GSC defect occurs in either single mutant (*lst-1* or *sygl-1*), but all GSCs are lost in early larvae of *lst-1 sygl-1* double mutants (Kershner et al., 2014). Consistent with a key role in self-renewal, both proteins are restricted to the GSC pool, and expanded expression of either LST-1 or SYGL-1 drives formation of a germline tumor (Shin et al., 2017). Thus, the stem cell function of LST-1 and SYGL-1 is equivalent to that of the self-renewal PUFs, i.e. they are responsible for GSC self-renewal throughout development.

The first clue that LST-1 and SYGL-1 might function together with PUF proteins in a complex came from a genetic finding that forced overexpression of LST-1 or SYGL-1 does not lead to tumor formation in the absence of FBF-1 and FBF-2 (Shin et al., 2017). Why might LST-1 and SYGL-1 depend on these two PUF proteins? LST-1 and SYGL-1 interact with FBF-1 and FBF-2 in yeast two-hybrid assays (Shin et al., 2017), and similarly interact with PUF-3 and PUF-11 (Boxem et al., 2008; Haupt et al., 2020; Qiu et al., 2019; Racher and Hansen, 2012). These findings crystallized the idea that LST-1 and SYGL-1 likely function as PUF partners. Consistent with that idea, an FBF target RNA, *gld-1*, is de-repressed in *lst-1 sygl-1* double mutants (Shin et al., 2017).

More recently, two PUF-interacting motifs (PIMs) were identified in the LST-1 amino acid sequence (Haupt et al., 2019). The ‘KxxL’ sequence of the LST-1 PIMs is similar to the ‘KTxL’ PIMs in other FBF partners, GLD-3 and CPB-1 (Campbell et al., 2012a,b; Menichelli et al., 2013; Wu et al., 2013). By yeast two-hybrid, an LST-1 protein with only one intact PIM can still bind PUF, but binding is lost when both were mutated. The biological impact of each LST-1 PIM (PIM-A and PIM-B) has been assayed in nematodes lacking SYGL-1, its redundant counterpart. Here again, LST-1 protein in which a single PIM is mutated, LST-1(A^m^) or LST-1(B^m^), retains its ability to maintain GSCs, but a double PIM mutant, LST-1(A^m^B^m^), does not (Haupt et al., 2019). Indeed, the two PIMs reside within a 210 amino acid ‘self-renewal region’ that harbors multiple IDRs and is both necessary and sufficient to maintain stem cells (Fig. 1F) (Haupt et al., 2019). *In vitro*, short LST-1 peptides carrying either of the two LST-1 PIMs bind to FBF-2 at the same site as CPB-1 and GLD-3 (Menichelli et al., 2013; Qiu et al., 2019, 2022; Wu et al., 2013). Together, these findings suggested that LST-1–PUF partnerships are important for stem cell regulation.

These earlier studies set the stage for testing the PUF hub model in nematodes and analyzing the molecular activities of self-renewal PUF partnerships. Here, we confirm that LST-1 physically associates with self-renewal PUF proteins in nematodes, but that a mutant lacking the LST-1 PIMs, LST-1(A^m^B^m^), does not. The LST-1(A^m^B^m^) mutant thus provides an incisive and unique tool to probe the functional significance of PUF partnerships *in vivo*. We demonstrate that LST-1 possesses repressive activity when tethered to a reporter RNA, and that its PUF partnership is essential for repression. We show further that LST-1 must partner with a PUF to associate physically with the CCR4-Not (CNOT) complex Based on these findings, we propose that the LST-1–PUF partnership is responsible for multiple molecular interactions that together form a stable effector complex on PUF target RNAs. Finally, we provide evidence that SYGL-1 functions much like LST-1 in GSC maintenance and RNA repression.

## RESULTS

### LST-1 associates *in vivo* with PUF proteins integral to the self-renewal hub

LST-1 and SYGL-1 are expressed at low levels in whole-worm extracts because of their spatial restriction to GSCs. We previously used a strong germline promoter to increase LST-1 and SYGL-1 abundance and managed to co-immunoprecipitate SYGL-1 with a single PUF protein, FBF-2 (Shin et al., 2017). However, that approach was technically challenging; it could not be extended to other PUF proteins for SYGL-1 and was unsuccessful for LST-1. To probe LST-1–PUF partnerships *in vivo*, we sought a different way to manipulate LST-1 levels. A conditional mutant of the GLP-1/Notch receptor, *glp-1(ar202)*, causes constitutive Notch signaling at restrictive temperature (25°C), expands the number of GSCs and drives formation of a germline tumor (Pepper et al., 2003); henceforth, we call this mutant *glp-1(gf ts)*. Because the *lst-1* gene is a direct target of GLP-1 signaling (Kershner et al., 2014; Lee et al., 2016), we expected that constitutive Notch signaling would expand LST-1 levels. We therefore generated a strain carrying *glp-1(gf ts)* and 3xV5 epitope-tagged LST-1 (LST-1^V5^). LST-1^V5^ was previously shown to retain wild-type LST-1 activity in stem cell regulation (Haupt et al., 2019). At the permissive temperature of 20°C, GSCs were maintained normally and LST-1^V5^ distribution appeared normal, but at restrictive temperature a germline tumor formed and LST-1^V5^ expanded to fill that tumor (Fig. 1C), but on a cell-by-cell basis LST-1 abundance in the tumors was comparable to regions of normal LST-1 expression in the distal gonad of non-tumorous strains. As reported previously (Pepper et al., 2003), small patches of differentiating cells were sometimes seen in the tumors, and LST-1^V5^ was missing from those patches (Fig. S1). Within germ cells, LST-1^V5^ was located in perinuclear granules and cytoplasm at both temperatures, as in non-tumorous germlines (Fig. 1D). We conclude that LST-1^V5^ retains normal activity, but that its expression becomes abundant with a simple shift to restrictive temperature in this easily maintained strain.

To determine whether the LST-1–PUF interactions found in yeast reflect interactions in nematodes, we generated a set of strains for co-immunoprecipitations (coIPs). Each strain carried *glp-1(gf ts)* and distinctly tagged LST-1 and PUF proteins (see Table S1 for specific genotypes). Control strains included *glp-1 (gf ts)* and each PUF tagged allele. LST-1^V5^ and LST-1^FLAG^ both functioned normally in genetic assays (Fig. 1E; Haupt et al., 2019), and similarly all tagged PUFs behaved normally (Fig. S2A). Moreover, all tagged PUFs were expressed throughout the germline at high levels in the tumors at restrictive temperature (Fig. S2B). Prior evidence for PUF hub partnerships relied on assays either *in vitro* or in yeast, all with protein fragments (Fig. S1D).

**Fig. 2.**
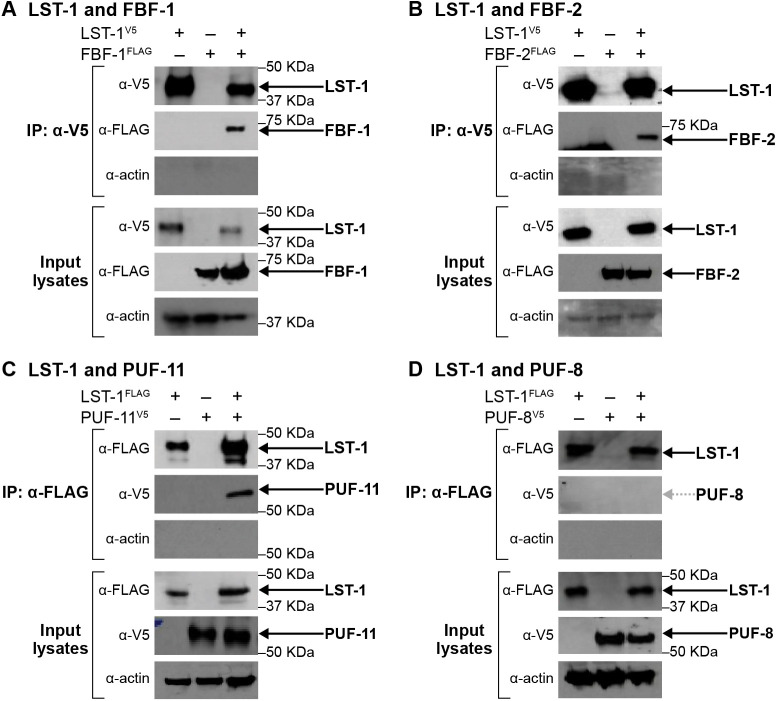
**LST-1 associates specifically with PUF hub proteins in nematodes.** (A-D) Western blots of input lysate and eluted samples after immunoprecipitation of epitope-tagged LST-1 from whole worms, after formaldehyde cross-linking. Blots were probed with relevant antibodies to detect epitope-tagged versions of LST-1, FBF-1 (A), FBF-2 (B), PUF-11 (C) and PUF-8 (D) as well as actin to see the loading control; 2% of input lysates and 20% of IP-eluted samples were loaded. Exposure times were different for input and IP lanes, so band intensities are not comparable. Arrows mark LST-1 and co-immunoprecipitated proteins. Gray dotted arrow indicates that PUF-8 did not co-immunoprecipitate with LST-1. Each coIP was repeated twice with similar results for the different replicates.

For the coIPs, we prepared lysate from at least 10^6^ synchronized adults with germline tumors; all animals were cross-linked with formaldehyde prior to collection. At least two replicates of each immunoprecipitation had similar results, both here and for other immunoprecipitations reported in this work. Among the PUF proteins in the self-renewal hub, LST-1 immunoprecipitation brought down FBF-1, FBF-2 and PUF-11 (Fig. 2A-C); PUF-3 was least abundant and not attempted. In contrast to the self-renewal PUFs in the hub, LST-1 did not co-immunoprecipitate with PUF-8, which is present in GSCs but is not essential for stem cell maintenance (Fig. 2D). Consistent with that finding, key residues in FBF-2, critical for LST-1 binding, are conserved in all four self-renewal PUFs, but not in PUF-8 (Qiu et al., 2019; Wu et al., 2013). We conclude that LST-1 associates in the nematode specifically with PUF proteins in the hub.

### ‘KxxL’ mutations abrogate LST-1–FBF interactions *in vivo*

**Fig. 3.**
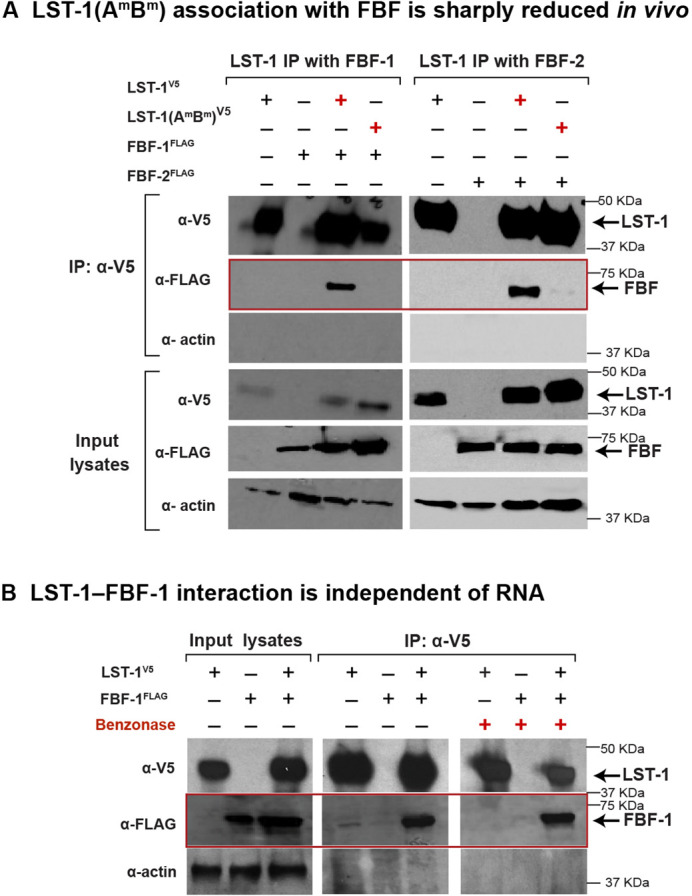
**LST-1–FBF interaction is PIM dependent and RNA independent.** (A) LST-1–FBF interaction requires LST-1 PIMs, PIM-A and PIM-B. Shown are western blots of input lysate and eluted sample after immunoprecipitation of epitope-tagged LST-1 from whole worms, after formaldehyde cross-linking. Blots were probed with anti-V5 antibody to see LST-1^V5^, anti-FLAG for FBF-1^FLAG^ and FBF-2^FLAG^, and anti-actin-4 for the loading control actin; 2% of input lysates and 20% of IP-eluted samples were loaded. Each coIP was repeated at least twice with similar results for different replicates. The red box highlights presence or absence of FBFs in the LST-1 immunoprecipitate. (B) LST-1–FBF interaction is independent of RNA. Shown are western blots of input lysate and eluted sample after immunoprecipitation of LST-1^V5^ from whole worms, without formaldehyde cross-linking and with or without Benzonase. Blots were probed as described in A; 2% of input lysates and 20% of IP-eluted samples were loaded. Each coIP was repeated twice with similar results for the different replicates. The red box highlights FBF-1 in the LST-1 immunoprecipitate.

Two PIMs in LST-1 mediate its PUF interactions in yeast (see Introduction). Here, we test the prediction that the PIM-defective LST-1(A^m^B^m^) mutant with 3XV5-epitope-tag [henceforth called LST-1(A^m^B^m^)^V5^ in the Results section] would not partner with PUF proteins in nematodes. To this end, we compared the ability of LST-1 or LST-1(A^m^B^m^)^V5^ protein to immunoprecipitate FBF- 1^FLAG^ or FBF-2^FLAG^ from worm lysates (Fig. 3A) using the protocol described in the previous section of ‘Results’ with animals cross-linked prior to collection. FBF-1 and FBF-2 were abundant in input lysates, and wild-type LST-1 successfully brought down both FBF-1 and FBF-2 (Fig. 3A, red box, third lane of each experiment). However, no detectable FBF-1 came down with LST-1(A^m^B^m^)^V5^ (Fig. 3A, red box, fourth lane of FBF-1 experiment), and FBF-2 was sharply reduced (Fig. 3A, red box, fourth lane of FBF-2 experiment). We conclude that the PIMs are indeed required for LST-1 association with PUF proteins in nematodes and that the LST-1(A^m^B^m^)^V5^ mutant abrogates that interaction.

We finally investigated whether the LST-1–PUF *in vivo* association depends on binding nucleic acid. In this case, worms were not subjected to formaldehyde cross-linking, and lysates were incubated prior to immunoprecipitation with Benzonase, an enzyme that cleaves both DNA and RNA. This experiment was performed with worms carrying wild-type LST-1^V5^ and FBF-1^FLAG^. The efficiency of FBF-1 recovery in LST-1 immunoprecipitations was similar with or without Benzonase (Fig. 3B). We conclude that LST-1 associates with FBF-1 independently of both DNA and RNA and suggest that this is likely true for other LST-1–PUF partnerships.

### Tethered LST-1 represses expression of a reporter RNA

**Fig. 4.**
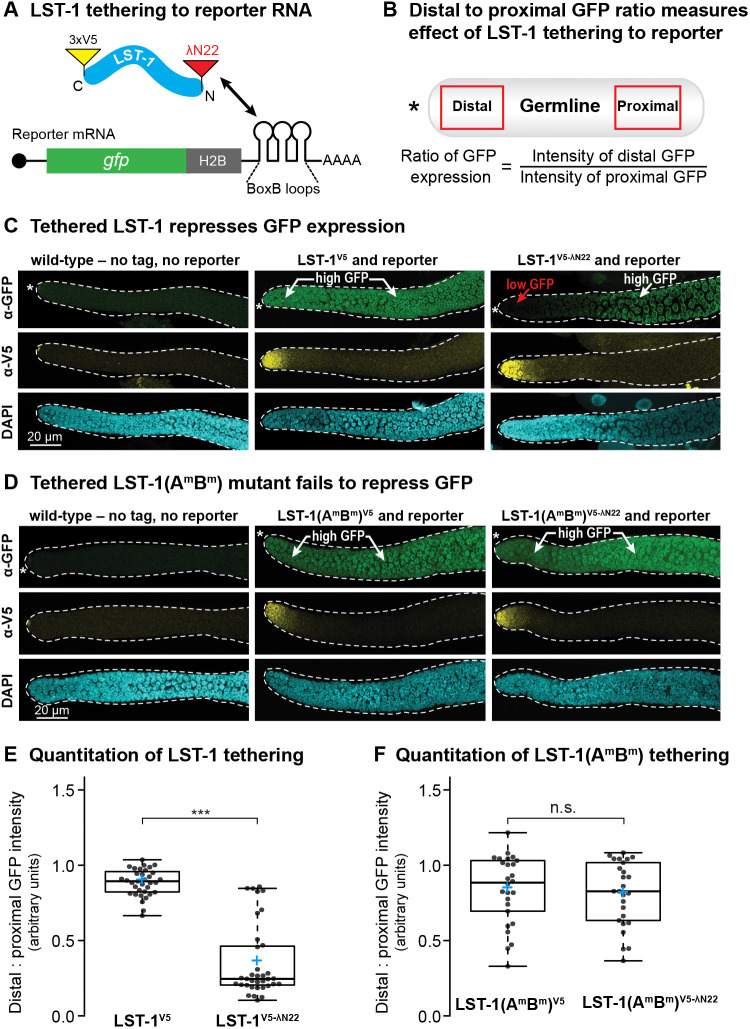
**LST-1 repressive activity is PIM dependent in tethering assay.** (A) Schematic of the tethering assay. LST-1^V5-λN^ carries a C-terminal V5 (yellow) and N-terminal λN22 (red). LST-1^V5-λN^ binds to BoxB hairpins for recruitment to reporter mRNA. (B) Quantitation of the effect of tethered LST-1 on reporter expression. GFP intensity was compared in the distal germline (1-40 µm from the DTC), where LST-1 is expressed at a high level, to GFP intensity more proximally (80-120 µm from the DTC), where LST-1 is expressed at a vanishingly low level. (C,D) Tethering results. Representative confocal images (maximum *z*-projection) of extruded gonads stained with anti-GFP (top) and anti-V5 (middle) antibodies and DAPI (bottom). GFP is green and LST-1 is yellow when tagged; DAPI marks all gonadal nuclei. An asterisk marks the distal end. (C) Tethering LST-1^V5-λN^. Left: control, no tag and no reporter; middle: untethered LST-1, V5 and reporter but no λN22; right: tethered LST-1, V5 and λN22 plus reporter. An asterisk marks the distal end. (D) Tethering LST-1(A^m^B^m^)^V5-λN^. Columns same as C. LST-1(A^m^B^m^)^V5^ and LST-1(A^m^B^m^)^V5-λN^ are both restricted to distal end; LST-1(A^m^B^m^)^V5-λN^ does not repress reporter expression. Asterisk marks distal end. Dashed lines mark gonad boundary. (E,F) Boxplots of distal:proximal GFP intensity ratios. Each dot represents a separate sample. Boxes represent 25th-75th quantile; middle line, median; blue plus sign, mean; whiskers, minimum and maximum values. ***P<0.0001 (two-tailed Student's *t*-test). n.s., not significant. (Difference between LST-1^V5^ and LST-1^V5-λN^: *P*=1.25×10^−18^; difference between LST-1(A^m^B^m^)^V5^ and LST-1(A^m^B^m^)^V5-λN^: *P*=0.63). Sample sizes: LST-1^V5^, *n*=35; LST-1^V5-λN^, *n*=35; LST-1(A^m^B^m^)^V5^, *n*=26; LST-1(A^m^B^m^)^V5-λN^, *n*=26.

Previous experiments suggesting that LST-1 and SYGL-1 have RNA repressive activity did not examine the two proteins individually and removed them genetically, which can lead to indirect effects (Shin et al., 2017). Here, we sought to test the regulatory activity of LST-1 directly and on its own. To this end, we adopted a protein–mRNA tethering strategy (Coller et al., 1998; Coller and Wickens, 2007). For tethering, we used the bacteriophage peptide λN22 and BoxB sites in RNA (Baron-Benhamou et al., 2004). We introduced λN22 at the N terminus of LST-1^V5^ and confirmed that the doubly tagged LST-1^V5-λN22^ protein is functional (Fig. S3A,B). Both LST-1 tags were inserted into the endogenous gene; the LST-1^V5^ and LST-1^V5-λN22^ proteins were similarly limited to the distal gonad *in vivo*. The reporter RNA, an integrated construct, relies on the strong germline *mex-5* promoter to drive transcription of a GFP–histone H2B RNA with three BoxB sites in its 3′UTR (Aoki et al., 2021, 2018; Baron-Benhamou et al., 2004) (Fig. 4A). This reporter RNA lacks a PUF-binding site.

To measure the effects of LST-1 on expression of the reporter, we compared GFP intensity in the region of the gonad where LST-1 was expressed at a high level (distal gonad) to the region where LST-1 was expressed at a vanishingly low level, just above background (more proximal in the gonad) (Fig. 4B). The ratio of distal to proximal GFP provides a quantitative measure of LST-1 RNA regulation and is internally controlled in each gonad. By GFP staining in individual gonads, the reporter was expressed in both distal and proximal regions when LST-1 was untethered (Fig. 4C, middle column), but tethered LST-1^V5-λN22^ protein lowered expression distally (Fig. 4C, right column). We conclude that LST-1 recruited to a reporter RNA represses expression.

We next investigated whether the LST-1–PUF interaction is required for repression. To this end, we inserted λN22 at the N terminus of the LST-1(A^m^B^m^)^V5^ mutant to generate a double-tagged LST-1(A^m^B^m^)^V5-λN22^ protein. When tested with the reporter, GFP staining was indistinguishable between untethered LST-1(A^m^B^m^)^V5^ and tethered LST-1(A^m^B^m^)^V5-λN22^ proteins (Fig. 4D, compare middle and right columns). Quantitation confirmed these tethering results for both wild-type LST-1^V5-λN22^ (Fig. 4E) and mutant LST-1(A^m^B^m^)^V5-λN22^ (Fig. 4F). We conclude that wild-type LST-1 possesses RNA repressive activity, but the mutant LST-1(A^m^B^m^)^V5^ does not. The LST-1–PUF partnership thus appears to be essential for repression.

### LST-1 associates with NTL-1 in nematode GSCs

Many RNA regulatory complexes recruit the CNOT complex to repress target mRNAs (Miller and Reese, 2012; Passmore and Coller, 2022). To determine whether LST-1 associates with the complex, we focused on NTL-1 (also known as LET-711), the *C. elegans* homolog of the Not1 scaffold protein (DeBella et al., 2006; Nousch et al., 2013). We first inserted three tandem FLAG tags at the C terminus of the endogenous *ntl-1* locus (Fig. S4A) and confirmed that NTL-1^FLAG^ was expressed throughout the germline (Fig. S4B), as seen previously for a different tagged version at the same site (Nousch et al., 2013). This NTL-1^FLAG^ protein retains biological function, as homozygous animals were viable and fertile (Fig. S4C).

**Fig. 5.**
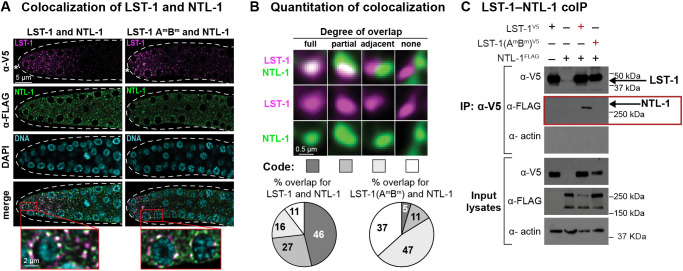
**LST-1 association with NTL-1 is PIM dependent.** (A) LST-1 colocalizes with NTL-1 *in vivo*. Representative deconvolved single confocal *z*-slices from middle plane of the distal region of an extruded gonad. Left: strain carrying both LST-1^V5^ and NTL-1^FLAG^; right: strain carrying both LST-1(A^m^B^m^)^V5^ and NTL-1^FLAG^. Row 1, V5 antibody detects LST-1 (magenta); row 2, FLAG antibody detects NTL-1 (green); row 3, DAPI highlights nuclei (cyan); row 4, merged images show co-staining with LST-1/NTL-1 overlap seen as white; insets, magnification of co-staining. Dashed line marks gonad boundary and asterisk marks distal end. (B) Variable colocalization of LST-1 and NTL-1. Images show representative examples of different degrees of overlap, taken from staining in A with further magnification. Code, shown below, is used in pie charts to show varying percentages of overlap with LST-1^V5^ (left) and LST-1 (A^m^B^m^)^V5^ mutant (right). Data in pie charts was generated from imaging ten gonads of each strain, with 200 LST-1 foci scored in the same region of each gonad (1-30 µm from the distal tip). (C) LST-1^V5^ and NTL-1^FLAG^ co-immunoprecipitation. Western blots were probed with V5 antibody for LST-1, FLAG antibody for NTL-1, and actin-4 antibody for the loading control; 2% of input lysates and 20% of eluted samples were loaded. Exposure times of input and IP lanes are different, so band intensities are not comparable. The coIPs were repeated twice with similar results for the different replicates. The red box highlights presence or absence of NTL-1 in the LST-1 immunoprecipitate.

We first investigated the LST-1–NTL-1 association by immunostaining. LST-1^V5^ and NTL-1^FLAG^ both reside in perinuclear puncta within GSCs (Haupt et al., 2019; Nousch et al., 2013; Shin et al., 2017). Co-staining revealed strong colocalization of wild-type LST-1^V5^ with NTL-1^FLAG^ (Fig. 5A). Most LST-1^V5^ and NTL-1^FLAG^ puncta overlapped fully or partially, with others that were adjacent or did not overlap (Fig. 5B). By contrast, most puncta with LST-1(A^m^B^m^)^V5^ did not overlap either fully or partially with NTL-1^FLAG^ puncta (Fig. 5A,B). This striking difference suggests that the LST-1–NTL-1 *in vivo* association relies on the LST-1–PUF partnership.

We next investigated whether LST-1^V5^ and NTL-1^FLAG^ co-immunoprecipitate from nematodes. The same protocol used for LST-1–PUF coIP (Fig. 2, Fig. 3) was followed, again with formaldehyde cross-linking. LST-1^V5^ did co-immunoprecipitate with NTL-1^FLAG^ (Fig. 5C, red box, third lane), but LST-1(A^m^B^m^)^V5^ did not (Fig. 5C, red box, fourth lane). The western blot for NTL-1 detected two major bands in the input lysates, one at ∼260 kDa and another one at ∼170 kDa, but only the larger band in the immunoprecipitate elutes. This larger band was similar in size to that detected previously using a LAP tag (Nousch et al., 2013). The smaller band may be a different isoform or a degradation product. The LST-1–PUF partnership thus appears to be essential for LST-1 association with the CNOT scaffold protein.

### SYGL-1 possesses two PUF-interacting motifs and has RNA repressive activity

**Fig. 6.**
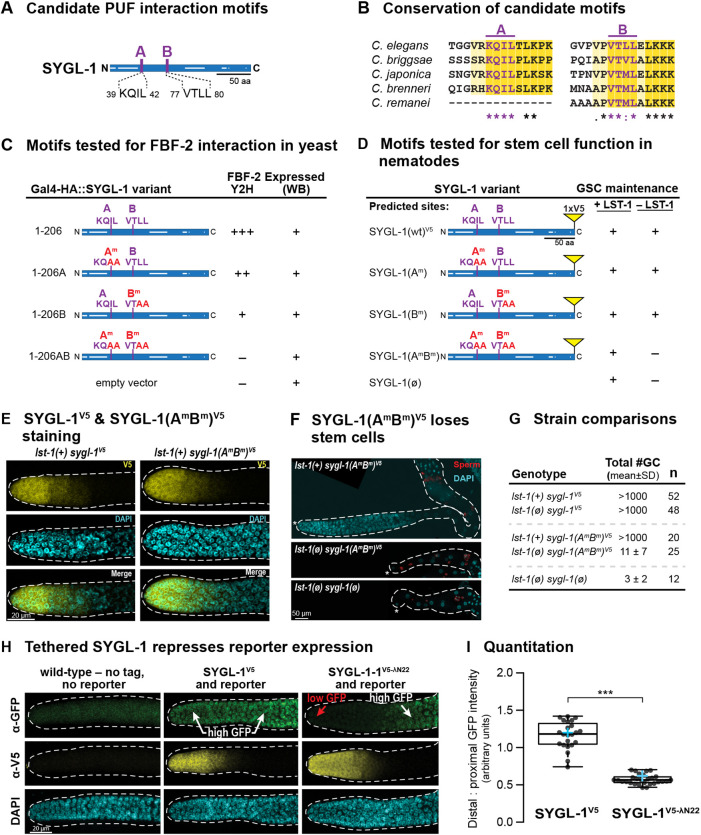
**SYGL-1 has two PUF-interacting motifs and RNA repressive activity.** (A) Diagram of SYGL-1 with its multiple IDRs (white lines internal to and along the axis of the rectangle representing the protein). Two candidate PIMs, PIM-A and PIM-B, were identified in the SYGL-1 amino acid sequence. (B) Conservation of PIM-A and PIM-B in SYGL-1 orthologs from related *Caenorhabditid* species. (C) Summary of SYGL-1 PIM effects on FBF binding, yeast two-hybrid assay. Superscript m denotes a mutant, with amino acid changes in red. +++, strong binding; ++, weaker binding; +, poor binding; −, no binding. (D) Summary of SYGL-1 PIM effects on GSC maintenance in nematodes. Mutation conventions as in C. SYGL-1 self-renewal activity was scored both in the presence of its LST-1 redundant counterpart as a control and in the absence of LST-1. (E) Spatial restriction of SYGL-1^V5^ and SYGL-1(A^m^B^m^)^V5^ to distal gonad. Representative confocal *z*-projections of extruded gonads stained with V5 antibody (yellow) and DAPI (cyan). Dashed line marks gonad boundary. (F) SYGL-1(A^m^B^m^)^V5^ has lost self-renewal activity. Representative *z*-projected confocal images of extruded gonads stained with SP56 antibody (red) for sperm and DAPI (cyan). Dashed line marks gonad boundary and asterisks mark the distal end. Top: in the presence of wild-type LST-1, SYGL-1(A^m^B^m^)^V5^ has no effect on GSC self-renewal; middle: in the absence of LST-1, SYGL-1(A^m^B^m^)^V5^ cannot maintain GSCs because the germline is tiny and GSCs differentiated in early larvae to produce a few sperm: bottom: *lst-1(ø) sygl-1(ø)* germlines are similar to *lst-1(ø) sygl-1(A^m^B^m^)^V5^* germlines. (G) Number of total germ cells (GC) per animal in different strains. Total number of germ cells in *lst-1(ø) sygl-1(A^m^B^m^)^V5^* is more than *lst-1(ø) sygl-1(ø),* but fewer than *lst-1(+) sygl-1(A^m^B^m^)^V5^*. (H) Tethered SYGL-1 reveals RNA repressive activity. Assay is same as in Fig. 4A, except λN22 is inserted at the C terminus of SYGL-1^V5^. Images show representative *z*-projection of distal region in extruded gonads. Dashed line marks gonad boundary. Top: anti-GFP antibody detects GFP; middle, anti-V5 detects SYGL-1; bottom, DAPI highlights DNA within gonadal nuclei. Left: control, no tag and no reporter; middle: untethered SYGL-1, V5 and reporter but no λN22; right: tethered SYGL-1, V5 and λN22 plus reporter. (I) Boxplots of distal:proximal GFP intensity ratios. Conventions as described in Fig. 4E,F. ***P<0.0001 (two-tailed Student's *t*-test). *P*=1.296×10^−14^ between SYGL-1^V5^ and SYGL −1^V5-λN^. Sample sizes: SYGL-1^V5^, *n*=23; SYGL-1^V5-λN^, *n*=28.

LST-1 and SYGL-1 are functionally redundant for stem cell regulation (see Introduction), but little was known about the similarity of their molecular functions. We first investigated whether SYGL-1 possesses PUF-interacting motifs. Two candidate motifs in the SYGL-1 amino acid sequence (Fig. 6A) were conserved in orthologs (Fig. 6B). Previous studies with other PUF partners highlighted the fourth leucine as most important (Menichelli et al., 2013; Wu et al., 2013), and crystal structures of FBF-2 with each of the LST-1 PIMs also highlighted the terminal leucine in the signature motif (Qiu et al., 2019, 2022). We therefore mutated those leucines to alanines in the candidate PIMs of SYGL-1, and also mutated their N-terminal neighboring amino acid (Fig. 6C). By yeast two-hybrid, wild-type SYGL-1 interacted well with both FBF-1 and FBF-2, SYGL-1 mutants defective in a single PIM lowered that interaction significantly, and mutants defective in both PIMs abolished it (Fig. 6C; Fig. S5C). The FBF-binding strengths of the two PIMs in yeast were distinct, with PIM-A weaker than PIM-B (Fig. S5C). A similar disparity was seen for the two PIMs in LST-1 (Haupt et al., 2019). Moreover, locations of the two PIMs were similar in LST-1 and SYGL-1. LST-1(PIM-A) and SGYL-1(PIM-A) begin at 32 and 39 amino acids from the N terminus, respectively, and LST-1(PIM-B) and SGYL-1(PIM-B) begin at 80 and 77 amino acids from the N terminus, respectively. This similarity in PIM number and spacing likely relates to the geometry of their PUF binding in a way we do not yet understand. Regardless, we conclude that SYGL-1 possesses two PUF-interacting motifs.

To determine whether the SYGL-1 PIMs affect stem cell function, we edited the key residues in *C. elegans* (Fig. 6D)*.* We did so in a previously edited endogenous locus that encodes a fully functional V5-tagged SYGL-1 protein. We thus generated SYGL-1(A^m^)^V5^ and SYGL-1(B^m^)^V5^ single mutants and a SYGL-1(A^m^B^m^)^V5^ double PIM mutant. All SYGL-1 variants were fertile in the presence of wild-type LST-1. In the absence of LST-1, the single PIM mutants retained their ability to maintain GSCs, demonstrating that one PIM is sufficient for SYGL-1 biological activity. However, the double PIM mutant was unable to maintain stem cells in the absence of LST-1 (Fig. 6D), despite SYGL-1 (A^m^B^m^)^V5^ protein being expressed in GSCs (Fig. 6E). In *lst-1(ø) sygl-1(A^m^B^m^)* mutants, all GSCs differentiated at an early larval stage (Fig. 6F,G). We conclude that the two SYGL-1 PUF-interacting motifs are essential for stem cell maintenance.

Finally, we tested SYGL-1 for its ability to repress expression of the GFP::H2B reporter when tethered, using the assay explained above for LST-1. To this end, we inserted the λN22 peptide at the SYGL-1 C terminus in the endogenous locus encoding wild-type SYGL-1^V5^. We thus generated a double-tagged SYGL-1^V5-λN22^, which retains its wild-type ability to maintain stem cells in the absence of LST-1 (Fig. S5D). Because SYGL-1 protein is restricted to the distal germline (Fig. 6H, middle row), we quantified its ability to repress the reporter by measuring the ratio of distal GFP to proximal GFP, as explained for LST-1 (Fig. 4B). The tethered protein SYGL-1^V5-λN22^ substantially lowered GFP expression in the distal gonad (Fig. 6H,I). Thus, SYGL-1^V5-λN22^ has repressive activity. We were unable to generate a PIM-defective SYGL-1^V5-λN22^, despite considerable effort. We conclude that SYGL-1 shares two key molecular properties with LST-1: possession of two PUF-interacting motifs essential for stem cell maintenance and the ability to repress expression of an RNA when tethered.

## DISCUSSION

### Understanding PUF hub partnerships through the lens of LST-1

**Fig. 7.**
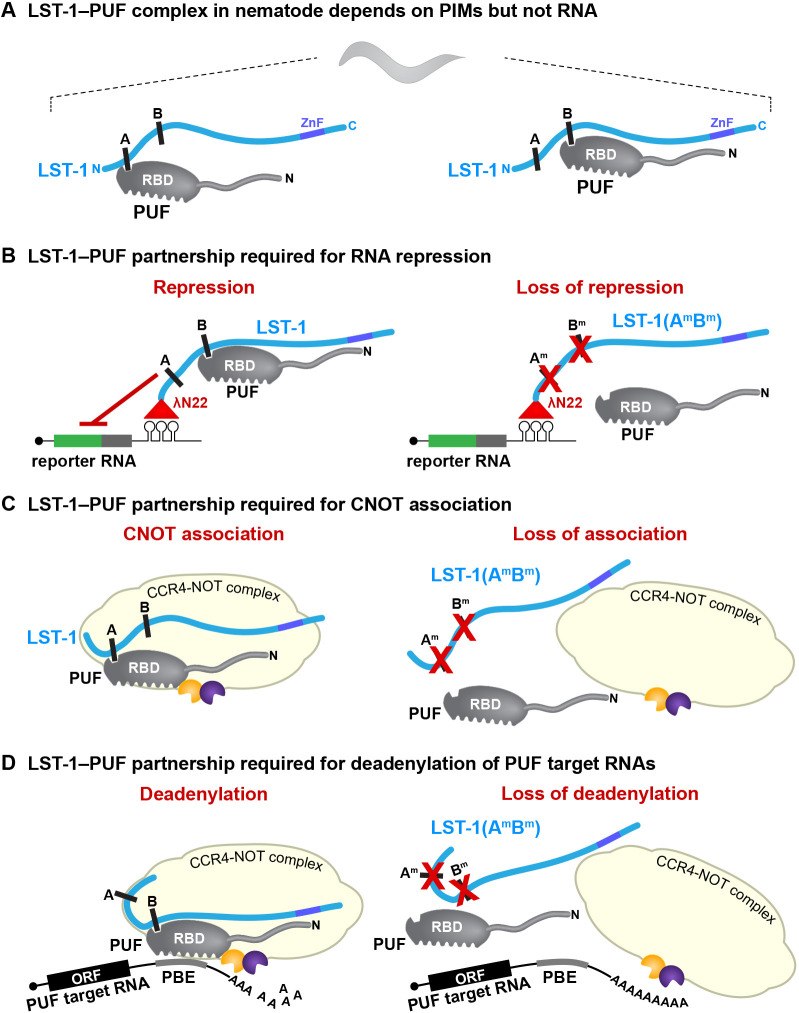
**Models for assembly and function of LST-1–PUF partnership in nematodes.** (A) Assembly of LST-1–PUF partnerships. LST-1 (blue) can bind to PUF proteins (gray) via either of two PIMs (A and B) (Haupt et al., 2019). No RNA is depicted because LST-1–PUF assembly does not require RNA (this work). PUF proteins comprise an RNA-binding domain (RBD) and an N-terminal tail (wavy line) with IDRs (Fig. S6). LST-1 is largely intrinsically disordered (wavy line) and also has C-terminal zinc finger (ZnF; purple); LST-1 stem cell function resides in its IDR region and does not require the zinc finger. Conventions in A are also used in B-D. (B) LST-1 RNA repressive activity depends on PUF partnership. When tethered (left), the LST-1–PUF complex represses expression of the reporter RNA; a PUF protein is included in this diagram, because PIM-defective LST-1(A^m^B^m^) cannot repress reporter RNA expression when tethered (right). Tethering employs λN22 (red triangle) fused to LST-1 to bind BoxB stem loops in reporter RNA (see Fig. 4A). (C) LST-1–CNOT association depends on PUF partnership. Left: LST-1–PUF complex associates with CNOT complex (light yellow). Right: PIM-defective LST-1(A^m^B^m^) disrupts the LST-1–PUF complex and destabilizes CNOT association. Dark yellow and blue shapes represent CCF-1 and CCR-4 deadenylases. The model includes an interaction between the PUF protein and CCF-1, based on work with FBF-1 and FBF-2 (Suh et al., 2009). (D) The LST-1–PUF partnership brings together multiple interactions to form a stable complex with the CNOT complex and repress target RNAs. Left: the LST-1–PUF complex provides LST-1 IDRs and PUF IDRs; PUF protein contacts CCF-1 and also binds the PUF-binding element (PBE) in its target RNA. Right: PIM-defective LST-1(A^m^B^m^) disrupts LST-1–PUF and destabilizes the larger complex (LST-1–PUF–CNOT–target RNA), shown here as loss of all interactions for simplicity.

The ‘PUF hub’ of the *C. elegans* GSC regulatory network provides a powerful entrée for analyzing the functional significance of partnerships between PUF RNA-binding proteins and their modulating partners (see Introduction; Fig. 1A,B). Here, we test key elements of the PUF hub model in nematodes for the first time and investigate how the PUF partnerships regulate GSCs. We focus on LST-1–PUF as a paradigm, because a nematode mutant had been created with potential to assess the *in vivo* function of LST-1–PUF partnerships. This LST-1(A^m^B^m^) mutant lacks amino acid residues responsible for its PUF interaction in yeast (Haupt et al., 2019). We confirm in this work that wild-type LST-1 associates with PUF hub proteins in nematodes, but that the LST-1(A^m^B^m^) mutant protein does not. Therefore, assembly of LST-1–PUF partnerships in nematodes depends on PIMS, as predicted. However, the LST-1–PUF complex did not depend on RNA. Because each PIM can act independently to bind PUFs in yeast and to promote GSC self-renewal in worms (Haupt et al., 2019; Qiu et al., 2019, 2022), our model for LST-1–PUF assembly includes two distinct complexes, one anchored by PIM-A (Fig. 7A, left) and the other by PIM-B (Fig. 7A, right), but not an RNA. We conclude that LST-1 forms PIM-dependent but RNA-independent partnerships with PUF hub proteins in the nematode (Fig. 7A).

Previous experiments implicated LST-1 and SYGL-1 in RNA repression (Shin et al., 2017). However, those experiments did not test the two proteins separately; they did not test significance of the PUF partnership; and they did not identify the likely effector of RNA repression. This work tackles all three issues and does so in nematode GSCs – the natural context. We used a tethering assay to investigate LST-1 RNA regulatory activity. This assay is direct, and it queries LST-1 separately from SYGL-1. The tethered LST-1 dramatically lowered expression of the reporter RNA. A common interpretation of this result would be that LST-1 acts alone to repress RNA. However, we also tethered the LST-1(A^m^B^m^) mutant, which cannot assemble an LST-1–PUF complex. To our surprise, LST-1(A^m^B^m^) lost RNA repressive activity. Although LST-1 PIMs might mediate binding to some non-PUF protein, the PIM-dependent PUF interaction is remarkably specific (self-renewal PUF proteins only), and we favor the simpler explanation – that LST-1 retains its partnerships with PUF proteins when recruited to the reporter RNA (Fig. 7B). How might an LST–PUF partnership repress RNA? PUF proteins recruit the CNOT complex to their target RNAs in virtually all eukaryotes – from yeast and plants to flies and humans (Nishanth and Simon, 2020). Nematode FBF-1 and FBF-2, for example, interact physically with a subunit of the CNOT complex, the nematode homolog of the CAF1 deadenylase, and they also promote its enzymatic activity *in vitro* (Suh et al., 2009). Here, we show that LST-1 colocalizes in subcellular puncta with the nematode homolog of the Not1 scaffold protein of the CNOT complex, called NTL-1 (Nousch et al., 2013), and, in addition, that LST-1 co-immunoprecipitates with NTL-1. However, the PIM-defective LST-1(A^m^B^m^) mutant dramatically reduces colocalization and does not co-immunoprecipitate with NTL-1, suggesting that LST-1 repressive activity and LST-1–NTL-1 association both depend on the LST-1–PUF partnership (Fig. 7C).

Why is the LST-1–PUF partnership required for RNA repression and association with CNOT? Answering that question in molecular detail will require future experiments to analyze formation of a larger LST-1–PUF–CNOT–RNA complex and map the key interaction surfaces. However, this work, together with results from others, suggests the model that LST-1–PUF provides multiple interactions that work together to form a stable effector complex on PUF target RNAs (Fig. 7D). One type of interaction relies on regions predicted to be IDRs. In yeast, fly and human PUF proteins, IDRs associate with CNOT and enhance its deadenylase activity (Arvola et al., 2020; Enwerem et al., 2021; Webster et al., 2019) with longer IDRs enhancing better than shorter ones (Webster et al., 2019). The PUF IDRs are located in N-terminal ‘tails’ – variably long extensions N-terminal to the PUF RNA-binding domain. The fly Pumilio N-terminal tail harbors an IDR-rich RD3 domain that interacts specifically with the Not1, Not2 (also known as Rga) and Not3 subunits of the CNOT complex (Haugen et al., 2022). Nematode PUF proteins also have IDR-rich N-terminal tails (Fig. S6), but the tails are short (∼120 amino acids in worms versus ∼1000 in flies and ∼800 in humans). The LST-1–PUF partnership therefore brings a PUF protein with its N-terminal IDRs and LST-1 with its IDRs in the ‘self-renewal region’ (Fig. 1B) into a single complex (Fig. 7A). It seems likely that PUF and LST-1 IDRs work together to facilitate association with CNOT (Fig. 7D, left). A second interaction, seen *in vitro*, occurs between the FBF-1 and FBF-2 RNA-binding region and one CNOT subunit, the CCF-1/CAF deadenylase enzyme (Suh et al., 2009). FBF-1 and FBF-2 physically interact with CCF-1 and promote its deadenylase activity. A third interaction occurs between the PUF protein and its PUF-binding element in the target RNA. We suggest that these interactions together form a stable effector complex that promotes deadenylation and represses target RNAs (Fig. 7D, left). We also suggest that the effector complex is destabilized without the LST-1–PUF complex (Fig. 7D, right), with the diagram suggesting loss of all interactions for simplicity.

### SYGL-1 and LST-1 share molecular features that are crucial for PUF hub function

SYGL-1 and LST-1 are redundant for stem cell maintenance, but their amino acid sequences bear no similarity to each other. However, both consist largely of IDRs. Although LST-1 has a C-terminal zinc finger, its stem cell function resides in the IDRs, not the zinc finger (Haupt et al., 2019). A key question has been whether the IDRs of SYGL-1 and LST-1 employ similar molecular mechanisms. This work identifies two common molecular features, suggesting that they do.

First, SYGL-1 and LST-1 both possess two PIMs that function independently to maintain stem cells. Thus, SYGL-1 retains its self-renewal capacity when either PIM is mutated, but loses it when both are defective; the same is true of the two LST-1 PIMs (Haupt et al., 2019; this work). We do not know why LST-1 and SYGL-1 have two PIMs when a single PIM is sufficient for stem cell maintenance. In yeast two-hybrid assays and *in vitro*, the two LST-1 PIMs differ in their FBF-binding affinity, with PIM-A weaker than PIM-B (Haupt et al., 2019; Qiu et al., 2019, 2022); the same is true of the two SYGL-1 PIMs in yeast (this work). However, those different affinities may not be relevant in nematodes if mitigated by multiple interactions of the LST-1–PUF partnership with the CNOT complex. Previous work discovered a KTxL signature for PIMs in CPB-1 and GLD-3 (Menichelli et al., 2013; Wu et al., 2013). Identification of the LST-1 PIMs revealed a related KxxL sequence (Haupt et al., 2019; Qiu et al., 2019), which was also found in SYGL-1 PIM-A (this work). The SYGL-1 PIM-B sequence (VTLL), however, reduces the consensus PIM motif to a single leucine (this work). That leucine is critical for PUF binding in a spectrum of *in vitro* and *in vivo* assays, but additional molecular features must exist to provide context for its binding (Qiu et al., 2022). With the range of PIM sequences now available and others likely to emerge, it should be possible to determine whether all FBF partners have two PIMs and how *in vitro* PIM differences affect *in vivo* function.

The second molecular feature common to LST-1 and SYGL-1 is their RNA repressive activity when tethered. PUF proteins are well known for RNA repression, and above we discuss how the LST-1–PUF complex is required for repression of target mRNAs (Fig. 7D). Here, we suggest that the SYGL-1–PUF complex likely uses the same mechanism to recruit CNOT to PUF target mRNAs and repress them.

### LST-1–PUF and Nos–Pum partnerships: similarities and differences

*Drosophila* Nanos and Pumilio provide a well-established paradigm for interaction of a PUF protein with its partner (Arvola et al., 2017). Our growing understanding of the *C. elegans* LST-1–PUF partnerships invites comparison. The fly Nos–Pum and worm LST-1–PUF complexes share several features. Both regulate GSCs (Forbes and Lehmann, 1998; Shin et al., 2017); both repress RNAs (Sonoda and Wharton, 1999; Wang et al., 2020; this work); and both recruit the CNOT complex (Kadyrova et al., 2007; this work). In addition, Nanos and LST-1 both interact with their respective PUF proteins in the same molecular region – at the loop between 7th and 8th repeats of the RNA-binding domain (Qiu et al., 2019, 2022; Weidmann et al., 2016). And finally, spatial restriction of both Nanos and LST-1 is responsible for localizing PUF function to a specific region. Nanos localization in the posterior embryo restricts PUF-dependent RNA repression to that region, and LST-1 localization to the distal-most gonad restricts PUF-dependent RNA repression to the GSC pool. Importantly, the PUF protein distribution extends well beyond that of its partner for both Nos–Pum and LST-1–PUF complexes. Therefore, one might think *a priori* that LST-1 would be analogous to Nanos and that their PUF partnerships would function similarly.

Yet that simple idea seems to be wrong. One difference is obvious from their distinct use of zinc fingers. The two zinc fingers in Nanos are integral to its ternary complex with Pumilio and RNA (Curtis et al., 1997; Lehmann and Nusslein-Volhard, 1991; Weidmann et al., 2016). These Nanos zinc fingers bind RNA just upstream of the Pumilio response element and contribute to a molecular clamp that strengthens Pumilio binding to RNA (Weidmann et al., 2016). By contrast, the single Nanos-like zinc finger in LST-1 can be deleted without affecting LST-1–PUF regulation of stem cells (Haupt et al., 2019). Furthermore, SYGL-1, the redundant counterpart of LST-1, does not possess a zinc finger, underscoring the irrelevance of the zinc finger to the partnership. A second difference emerges from biochemical experiments testing how the partners affects PUF affinity for RNA. Nanos enhances PUF affinity for RNA (Weidmann et al., 2016), but PIM-bearing fragments of LST-1 weaken it (Qiu et al., 2019, 2022). This LST-1 conclusion may be misleading, however, given its reliance on a peptide with a single PIM. It will be important to learn whether full-length LST-1 strengthens or weakens PUF affinity for RNA. A third difference is that Nanos cannot bind stably to Pumilio without RNA (Arvola et al., 2017; Sonoda and Wharton, 1999; Weidmann et al., 2016), but LST-1 binds to FBF independently of RNA (this work). And finally, tethered Nanos represses RNA on its own (Raisch et al., 2016), but LST-1 does not repress RNA on its own – it needs its PUF partnership for repression (this work). Thus, the Nos–Pum and LST-1–PUF complexes represent two distinct modes of PUF partnership, despite both having a repressive RNA regulatory activity that relies on the CNOT complex. Those distinct modes showcase the LST-1–PUF partnership as an emerging paradigm for understanding how PUF proteins are modulated by partners to control gene expression.

## MATERIALS AND METHODS

### Strain maintenance

*C. elegans* strains were maintained as described (Brenner, 1974) at 20°C except those with *glp-1(gf ts)*, which were maintained at 15°C but shifted to 20°C or 25°C for experimentation. For a complete list of strains used in this study, see Table S1.

### RNA interference

RNA interference (RNAi) was performed by feeding, as described (Kershner et al., 2014; Timmons and Fire, 1998). *sygl-1* and *lst-1* clones from the Ahringer RNAi library (Fraser et al., 2000) were used for RNAi treatment, and L4440 plasmid (‘empty’ RNAi) for the negative control. For a detailed protocol, see Kershner et al. (2014).

### CRISPR/Cas9-induced allele generation

CRISPR/Cas9 genome-editing methods were used to alter endogenous *lst-1*, *sygl-1* and *ntl-1* genes (Table S1) using ribonucleoprotein complexes with a co-conversion strategy following an established protocol (Arribere et al., 2014; Paix et al., 2015). For a detailed protocol, see Haupt et al. (2019). Sequences of all crRNA (IDT) and DNA templates (IDT) are listed in Table S2.

### Scoring tumor production using DAPI staining

Germline tumors induced by *glp-1(ts gf)* at 25°C were scored with DAPI (4′,6-diamidino-2-phenylindole) staining of extruded gonads, following the protocol described by Crittenden et al. (2017), with some modifications. Briefly, animals were dissected in PBStw [PBS+0.1% (v/v) Tween-20] with 0.25 mM levamisole to extrude gonads, then fixed at room temperature for at least 15 min in 2% paraformaldehyde diluted in PBStw. Samples were incubated overnight at −20°C in 100% methanol. Next day, the samples were washed with PBStw, then incubated with 0.5 ng/μl DAPI in PBStw to label DNA. Then, samples were mounted in either Vectashield (Vector Laboratories) or ProLong Gold (Thermo Fisher Scientific). Tumors were confirmed by observation of proliferation throughout the germline, including metaphase plates proximally, and few, if any, gametes. Some germlines had patches of meiotic cells as previously described (Pepper et al., 2003).

### Immunostaining, microscopy, fluorescence quantitation

Immunostaining was performed as described (Crittenden et al., 2017) with minor modifications. Briefly, animals were staged to 24 h past mid-L4 stage (when grown at 20°C) or 18 h past mid-L4 stage (when grown at 25°C). Staged animals were dissected in PBStw (PBS+0.1% (v/v) Tween-20) with 0.25 mM levamisole to extrude gonads after cutting behind pharynx. Tissues were fixed in 3% (w/v) paraformaldehyde diluted in 100 mM K_2_HPO_4_ (pH 7.2) for 20 min. After fixation, all samples were permeabilized with ice-cold methanol (for worms that harbor GFP) for 20 min or PBStw+0.2% (v/v) Triton X-100 for 5-10 min. Samples were then washed twice by adding PBSTw followed by centrifugation at 1500 rpm (0.4 ***g***) for 60 s, then excess liquid was removed. Next, they were blocked with either 30% (v/v) goat serum diluted in PBStw (for anti-FLAG) or 0.5% (w/v) bovine serum albumin diluted in PBStw (all other antibodies) for 1 h. Primary antibodies were then added and samples incubated overnight at 4°C in blocking solution at the following dilutions: mouse anti-FLAG (1:1000, M2 clone, Sigma-Aldrich, F3165), mouse anti-V5 (SV5-Pk1, 1:1000, MCA1360, Bio-Rad) and mouse anti-SP56 (1:200, a gift from Susan Strome, University of California, Santa Cruz, CA, USA). Samples were washed twice the next day with PBSTw. For secondary antibodies, samples were incubated for 1 h at room temperature in dark at the following dilutions: donkey Alexa 555 anti-mouse (1:1000, Invitrogen, A31570), donkey Alexa 647 anti-mouse (1:500, Invitrogen, A31571). To visualize DNA, DAPI was added at 0.5-1 ng/μl during the last 20 min of secondary antibody incubation. Samples were then washed twice with PBSTw to remove excess antibodies. After the last wash, excess liquid was removed, and samples were mounted in ProLong Gold (Thermo Fisher Scientific) on microscope slides (12-544-1, Fisher Scientific) and covered with 22×22 coverslip (Marienfeld, ES0107052). Mounted samples were cured overnight before imaging. All images were taken using a laser-scanning Leica TCS SP8 confocal microscope with LASX software. Photomultiplier (PMT) detectors were used for DAPI and Hybrid (HyD) detectors were used for all other fluorescence. A 63×/1.40 CS2 HC Plan Apochromat oil immersion objective was used for all images, which were taken with the standard 400-700 Hz scanning speed and 100-300% zoom. Immunostaining quantitation was performed using Fiji/ImageJ. For a detailed protocol, see Haupt et al. (2019)

For the tethering assays, GFP intensity in the distal germline (1-40 µm from distal end) was compared with that more proximally [80-120 µm from the distal tip cell (DTC)] in the same germline using Fiji/ImageJ. Ratios of distal to proximal intensity were calculated using Microsoft excel software. Samples from at least three independent replicates were analyzed together after normalizing to a control with no GFP (N2).

### Progenitor zone count

Progenitor zone (PZ) size was scored in DAPI-stained extruded gonads from hermaphrodites 24 h past mid-L4 at 20°C or 18 h past mid-L4 at 25°C. PZ sizes were scored following the convention previously described (Crittenden et al., 2006; Seidel and Kimble, 2015). Scoring was done manually using the Fiji/ImageJ multi-point tool; each DAPI-stained nucleus along the edge of the tissue was considered a unique cell row; values from the two edges of the gonad were then averaged to determine PZ size.

### Immunoprecipitations and western blotting

*glp-1(gf ts)* animals were raised at 15°C or 20°C until they became gravid; they were then bleached to obtain synchronized offspring. Synchronized L1 worms were put at 25°C for 48 h to induce germline tumors. A minimum of 10^6^ young adults were collected using the following protocol: animals were washed twice with M9 buffer [3 g/l KH_2_PO_4_, 6 g/l NaHPO_4_, 5 g/l NaCl and 1 mM MgSO_4_] and cross-linked with 1% (w/v) formaldehyde for 10 min at room temperature. For the immunoprecipitations in Fig. 3B, samples were not cross-linked before collection. Worm pellets were then washed twice with M9 and snap-frozen in liquid nitrogen for subsequent analysis. Pellets were resuspended in 1 ml lysis buffer [20 mM Tris pH 7.5, 150 mM NaCl, 2 mM EDTA, 5 mM MgCl_2_, 1% (v/v) Triton X-100, 1 M urea, cOmplete Protease Inhibitor Cocktail (Roche)]. Urea was not used for samples processed to produce Fig. 3B. Worms were lysed by adding one sterilized Retsch 5-mm stainless steel ball to each sample, and then put in a Retsch 400 MM mill mixer at 4°C for three 10-min cycles at 30 Hz. After cycle 1 and 2, two 5-min freeze-thaw steps were performed by immersion in liquid nitrogen for 1 min followed by immersion in room-temperature water for 4 min. Lysates were cleared twice by centrifugation (16,000 ***g***, 15 min at 4°C), and the total protein concentration was measured using Bradford assay (Bio-Rad).

To prepare antibody-conjugated beads, 20 μg mouse anti-V5 (Bio-Rad, MCA1360) (for Figs 2A,B and 3A,B) or mouse anti-FLAG (M2 clone, Sigma-Aldrich, F3165) (for Fig. 2C,D) was incubated with 4.5 mg protein G Dynabeads (Novex, Life Technologies, 10003D) for 60 min at room temperature. The Dynabeads were then washed to remove unbound antibodies. The total amount of protein for immunoprecipitation was normalized to input. Twenty milligrams of lysates were incubated with the antibody-bead mixture for 4 h at 4°C, in the presence of RNase A at 10 μg/ml. RNA degradation was confirmed by isolating total RNA from post-immunoprecipitation lysates using TRIzol LS (Invitrogen, 10296028) and analyzing on agarose gels. For the immunoprecipitation in Fig. 3B, 1 µl of Benzonase^®^ Nuclease (Sigma Millipore, 250 U/µl) was used instead of RNase A for specific samples (see Fig. 3, legend). Beads were pelleted, washed four times with lysis buffer, and then two times with wash buffer [20 mM Tris pH 7.5, 0.5 M NaCl, 2 mM EDTA, 1% (v/v) Triton X-100]. Samples were then eluted with elution buffer [1% (w/v) SDS, 250 mM NaCl, 1 mM EDTA, 10 mM Tris pH 8] for 10 min at 100°C and analyzed by western blotting.

For western blotting, input and eluted samples were run on 10% acrylamide gel at room temperature. Then samples were transferred to PVDF membrane (Immobilon-P, 0.45 µm, Merck Millipore), which was activated prior to transferring in 100% methanol following a wash in ddH2O. The transfer was carried out for 4 h at 4°C in transfer buffer containing 20% methanol. For NTL-1 (Fig. 5), the transfer buffer contained 10% methanol to minimize precipitation of the large protein. After transfer, the membrane was blocked for 1 h at room temperature in 5% skimmed powdered milk. For primary antibodies, blots were incubated overnight at 4°C at the following dilutions: mouse anti-FLAG (1:1000, M2 clone, Sigma-Aldrich, F3165), mouse anti-V5 (1:1000, Bio-Rad, MCA1360), mouse anti-actin (1:40,000, C4 clone, Millipore, MAB1501). For secondary antibodies, blots were incubated for 1 h at room temperature with rat HRP-conjugated anti-mouse (1:10,000, Abcam, mAb 131368). To analyze the coIPs, blots were stripped with Restore™ Western Blot Stripping Buffer (Thermo Fisher Scientific). Immunoblots were developed using SuperSignal™ West Pico/Femto Sensitivity substrate (Thermo Fisher Scientific, 34080, 34095) and imaged using an ImageQuant LAS4000 (GE Healthcare). Fiji/ImageJ was used to adjust contrast. For each set of samples, coIPs were carried out at least twice.

### Colocalization assay

Colocalization (Fig. 5) was scored manually using Fiji/ImageJ. Confocal images of extruded gonads stained for DNA and both epitope-tagged LST-1 and NTL-1 were processed using the Leica Lightning deconvolution package. Composite images were used to score colocalization of LST-1 and NTL-1. LST-1 foci were first marked using multi-point tool in the distal 30 µm of the gonad using only the LST-1 channel. The NTL-1 channel was then added, and the degree of colocalization was manually scored. Two hundred LST-1 foci were scored in each gonad. The experiment was repeated twice with a total of 20 gonads for each strain.

### Yeast two-hybrid

Modified yeast two-hybrid assays were performed as described (Bartel and Fields, 1997). Briefly, *sygl-1* cDNA encoding wild-type, full-length SYGL-1 (aa 1-206), or full-length SYGL-1 carrying PIM mutations were cloned into the NcoI site in pACTII (Gal4 activation domain plasmid), generating pJK1580, pJK1581, pJK1582, pJK2094, pJK2095 and pJK2096 using the Gibson assembly method. The PUF repeat region of FBF-2 (aa 121-632) was cloned into the NdeI site in pBTMknDB (LexA-binding domain plasmid) to generate pJK2046. Activation and binding domain plasmids were co-transformed into the L40-ura strain using the Te-LiAc method (Gietz and Schiestl, 2007). *lacZ* reporter activity was assayed in defined media (SD) supplemented with -Leu-Trp using the Beta-Glow^®^ Assay System following commercially available protocols (Promega, E4720). In short, yeast cultures were grown to mid-log phase, diluted to the same optical density (0.1), and added to equal volumes of Beta-Glow^®^ reagent. Yeast clones were then incubated for 1 h at room temperature, and luminescence was quantitated using a Biotek Synergy 4 Hybrid plate reader and Gen5 software. A complete list of plasmids used in yeast two-hybrid assays is given in Table S3.

### Statistical analysis

Statistical analyses, sample sizes and *P*-values are described in figure legends. A two-tailed Student's *t*-test (T.TEST function in Microsoft excel) assuming equal-variance was performed when comparing two samples. A *P*-value less than 0.05 was considered significant. Box plots were generated with web tool BoxPlotR (Spitzer et al., 2014); quartiles and whiskers are indicated in legends.

## Supplementary Material

10.1242/develop.201705_sup1 Supplementary information
