# Supplementary material for: The in vivo functional significance of PUF hub partnerships in C. elegans germline stem cells
Source: Development. 2023 May 9;150(9):dev201705. doi: 10.1242/dev.201705 (PMC10259659; doi:10.1242/dev.201705)
Supplement: Supplementary information [file develop-150-201705-s1.pdf]

A LST-1^{FLAG} distribution in *glp-1(gf ts)* gonads

20ºC - permissive | **25ºC -- restrictive**

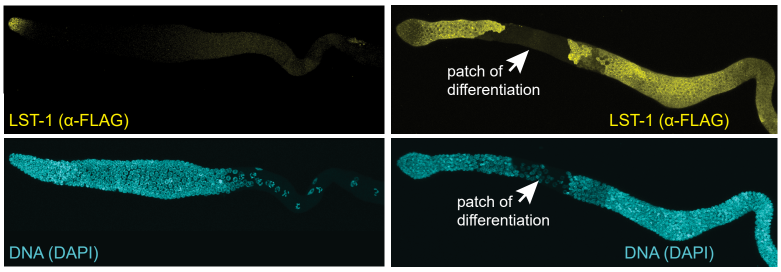

B LST-1(A^{m}B^m)^{V5} distribution in *glp-1(gf ts)* gonads that also carry FBF-2^{FLAG}

20ºC - permissive | **25ºC -- restrictive**

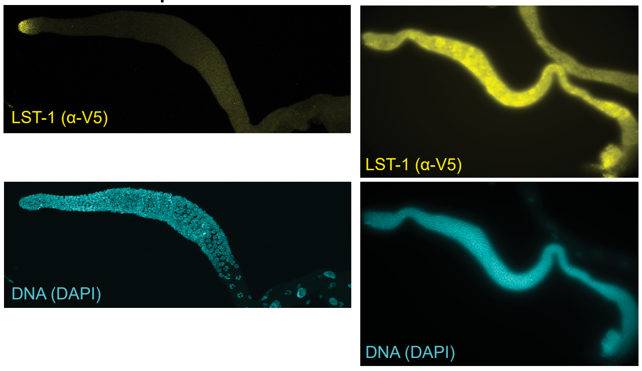

C. Phenotype characterization

Genotype*	% Fertility 20°C	n	PZ size mean gcd ± sd 20°C	n	% Tumor 25°C	n
lst-1^{V5}	100	100	19±1	10	0	50
lst-1^{V5}; glp-1(gf ts)	98	100	20±3	10	100	50
lst-1(A^{m}B^m)^{V5}	97	100	19±2	10	0	50
lst-1(A^{m}B^m)^{V5}; fbf-1^{FLAG}; glp-1(gf ts)	98	100	22±1	10	100	50
lst-1(A^{m}B^m)^{V5}; fbf-2^{FLAG}; glp-1(gf ts)	95	100	19±3	10	99	50

* See Table S1 for allele and strain numbers.
For Progenitor zone (PZ) size, n is number of gonadal arms scored.

D. Prior evidence for PUF hub partnerships

Partner	PUF	*in vitro*	Refs‡	yeast 2-hybrid	Refs‡
LST-1	FBF-1	no	n/a	yes	3
LST-1	FBF-2	yes	1, 2	yes	1, 2, 3
LST-1	PUF-3	no	n/a	no	n/a
LST-1	PUF-11	no	n/a	yes	3,4
SYGL-1	FBF-1	no	n/a	yes	3
SYGL-1	FBF-2	no	n/a	yes	3
SYGL-1	PUF-3	no	n/a	no	5
SYGL-1	PUF-11	no	n/a	no	n/a

‡ n/a, not applicable. 1 Qiu et al 2019; 2 Qiu et al 2022; 3 Shin et al., 2017; 4 Boxem et al 2008; 5 Haupt et al 2020.

Development • Supplementary information

Fig. S1. Supplementary analysis of LST-1 and LST-1($A^m B^m$) proteins.

A. Representative z-projection of extruded gonads showing LST-1^{FLAG} distribution at permissive and restrictive temperature in *glp-1 (gf ts)* animals.

B. Representative z-projection of extruded gonads showing LST-1($A^m B^m$)^{V5} distribution at permissive and restrictive temperature in *glp-1 (gf ts)* background.

C. Phenotypic characterization of lst-1($A^m B^m$) mutants. Fertility refers to self-fertility. PZ size refers to progenitor zone size, measured as the mean number of germ cell diameters (gcd) from the distal end to meiotic entry. The mean PZ size for wild-type is of 20 germ cell diameter (Crittenden et al., 2006). Tumor refers to a germline tumor.

D. Summary table of prior evidence for PUF hub partnerships. Ref, references.

Development • Supplementary information

A Phenotype characterization

Genotype *	% Fertility 20°C	n†	PZ size mean gcd ± sd 20°C	n†	% Tumor 25°C	n†	Reference
fbf-1^{FLAG}	100	112	19±2	10	0	100	this work
fbf-1^{FLAG} fbf-2(ø)	70§	133	24 ± 3	11	nd	n/a	this work
fbf-1^{FLAG}; glp-1(gf ts)	98	100	21±1	15	100	50	this work
lst-1^{V5}; fbf-1^{FLAG} ; glp-1(gf ts)	100	100	20±2	15	100	50	this work
fbf-2^{FLAG}	100	62	20±1	62	0	50	this work
fbf-1(ø) fbf-2^{FLAG}	100	53	15±2	53	n/a	n/a	this work
fbf-2^{FLAG}; glp-1(gf ts)	100	100	19±3	15	100	50	this work
lst-1^{V5}; fbf-2^{FLAG}; glp-1(gf ts)	100	100	21±1	15	100	50	this work
puf-11^{V5}	100	100	18±2	15	0	100	this work
puf-11^{V5}; glp-1 (gf ts)	100	100	21±2	15	100	50	this work
lst-1^{FLAG}; puf-11^{V5}; glp-1(gf ts)	100	100	19±1	15	100	50	this work
puf-8^{V5}	100	100	20±2	15	0	100	this work
puf-8^{V5}; glp-1(gf ts)	100	100	22±1	25	100	50	this work
llst-1^{FLAG}; puf-8^{V5}; glp-1(gf ts)	100	100	20±1	25	100	50	this work

* See Table S1 for allele and strain numbers.

† For % Fertile and % Tumor, n is number animals scored; for Progenitor zone (PZ) size, n is number of gonadal arms scored.

§ Non-fertile animals of this genotype make normal-sized germlines that are Fog (no sperm, oocytes only).

B Distribution of PUF proteins in *glp-1(gf ts)* worms at restrictive and permissive temperature

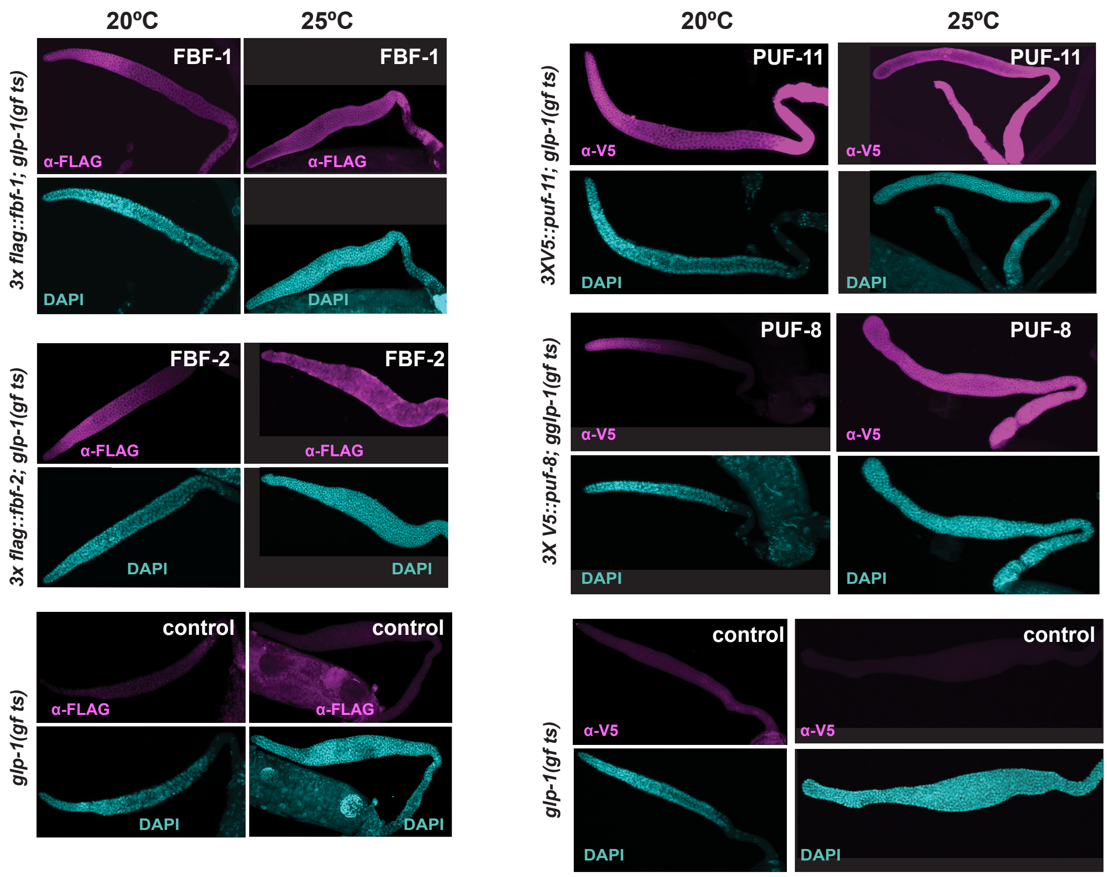

Fig. S2. Supplementary analysis of tagged PUF proteins.

A. Phenotypic characterization of animals carrying PUF proteins that were epitope tagged at the endogenous locus. Conventions as in Fig S1C.

B. Representative z-projection of extruded gonads showing distribution of PUF proteins at permissive temperature (20°C) and restrictive temperature (25°C).

Development • Supplementary information

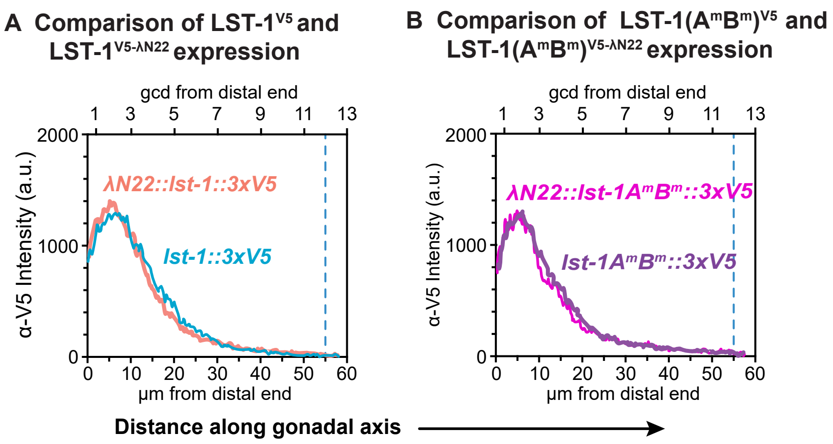

C LST-1$^{V5\text{-}\lambda N22}$ retains biological function

Genotype*		% Fertile 20°C	n†	PZ size mean gcd ± sd 20°C	n†
lst-1	sygl-1				
lst-1^{V5}	+	99	>100	20.0±2.0	10
lst-1^{V5}	ø	97	>100	13.2±1.5	15
lst-1$^{V5\text{-}\lambda N22}$	+	96	100	19.6±2.5	15
lst-1$^{V5\text{-}\lambda N22}$	ø	97	100	13.0±2.0	15
lst-1(A^{m}B^m)^{V5}	+	98	>100	20.0±1.0	15
lst-1(A^{m}B^m)^{V5}	ø	0	100	n/a	0
lst-1(A^{m}B^m)$^{V5\text{-}\lambda N22}$	+	98	100	21.0±2.0	15
lst-1(ø)	RNAi	0	100	n/a	0

* See Table S1 for allele and strain numbers.
† For % Fertile, n is number animals scored;
for Progenitor zone (PZ) size, n is number of gonadal arms scored.

Fig. S3. Supplementary analysis of LST-1$^{V5\text{-}\lambda N22}$ protein.

A. Expression: Left, LST-1^{V5} and LST-1$^{V5\text{-}\lambda N22}$ are expressed with similar abundance along axis of extruded gonads stained with α-V5. Right, LST-1(A^{m}B^m)^{V5} and LST-1(A^{m}B^m)$^{V5\text{-}\lambda N22}$ are expressed with similar abundance along axis of extruded gonads stained with α-V5.

B. Self-renewal activity: Conventions as in Fig S1C; brood size refers to total number of self-progeny produced by a single hermaphrodite. LST-1^{V5} and LST-1$^{V5\text{-}\lambda N22}$ both retain self-renewal activity; LST-1(A^{m}B^m)^{V5} and LST-1(A^{m}B^m)$^{V5\text{-}\lambda N22}$ both lose self-renewal activity.

Development • Supplementary information

A. Epitope tagging the endogenous *ntl-1* gene locus

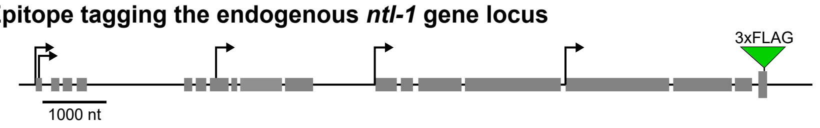

B. NTL-1 protein distribution in germline tumor

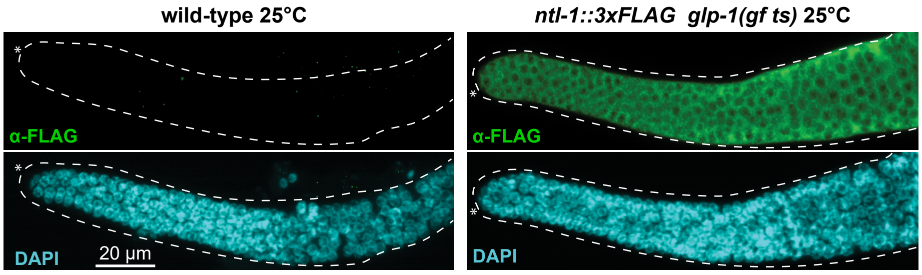

C. Phenotype characterization

Genotype*	% Fertility 20ºC	n	PZ size mean gcd ± sd 20ºC	n	Brood size mean ± sd 20ºC	n^	%Tumor 25ºC	n
wild-type	98	100	20±1	21	211±15	4	0	100
ntl-1^{FLAG}	99	100	19±2	25	220±13	4	0	100
ntl-1^{FLAG} *glp-1(gf ts)*	99	100	19±1	25	215±15	4	100	100
lst-1^{V5}; *ntl-1*^{FLAG} *glp-1(gf ts)*	97	100	19±2	30	220±10	4	100	100
lst-1$(A^mB^m)^{V5}$; *ntl-1*^{FLAG} *glp-1 (gf ts)*	97	100	20±2	20	210±10	4	100	100

[illegible] genitor zone (PZ size, n is number of gonadal arms scored.
[illegible] orms were scored for brood size;

Fig. S4. Supplementary analysis of NTL-1^{FLAG}.

A. Diagram of *ntl-1* gene shown to scale with all its alternative transcription start sites. The FLAG tag at the NTL-1 C-terminus labels all NTL isoforms.

B. Expression: Left, untagged NTL-1 is not detected; right, NTL-1^{FLAG} is expressed throughout the germline. Representative z-projected confocal images of extruded gonads stained using α-FLAG to detect NTL-1^{FLAG} (green) and DAPI to detect nuclei (cyan).

C. Phenotype: Conventions as in Fig S1C. At 20 °C, NTL-1^{FLAG} has no effect on Progenitor Zone Size, Brood Size or Fertility; at 25°C, NTL-1^{FLAG} has no effect on tumor formation.

Development • Supplementary information

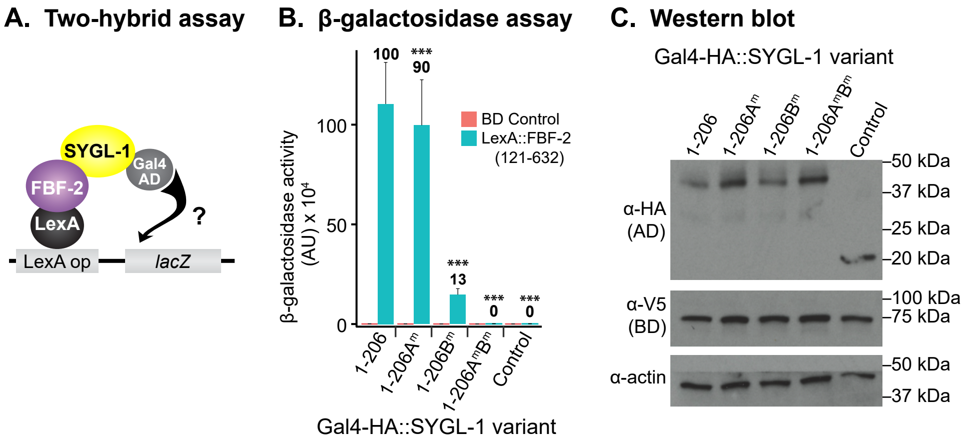

D. Both SYGL^{V5} and SYGL-1$^{V5\text{-}\lambda N22}$ function normally to maintain germline stem cells

Genotype	% Fertility	n	# PZ Size mean gcd ± sd	n
lst-1(+) sygl-1(+)	98	100	20 ± 2	20
lst-1(ø) sygl-1^{V5}	95	60	20 ± 1	20
lst-1(ø) sygl-1$^{V5\text{-}\lambda N22}$	94	81	19 ± 3	20
lst-1(ø) sygl-1(ø)	0	100	n/a	10

For Progenitor zone (PZ) size, n is number of gonadal arms scored.

Fig. S5. Supplementary SYGL-1 experiments

A. Schematic of yeast two-hybrid assay to test SYGL-1–FBF-2 interaction. Variants of full length SYGL-1(1-206) protein were fused to Gal4 activation domain (AD), and FBF-2(121-632), which includes all PUF repeats, was fused to LexA binding domain (BD). Interaction drives transcription of lacZ genes.

B. Results of β-gal assay. Each bar is an average of at least three independent replicates; error

bars show standard deviation. Activity for each variant is indicated as a percentage of the activity of wild-type SYGL-1, with the number above each bar. Asterisks indicate a statistically significant difference from wild-type by one-way ANOVA with Tukey's HSD test. ***, $p<0.001$; n.s., not significant ($p >0.05$).

C. Expression Gal4 fusion variants, assayed by Western blot.

D. Phenotype: Conventions as in Fig S1C. SYGL-1$^{V5\text{-}\lambda N}$ retains self-renewal activity.

Development • Supplementary information

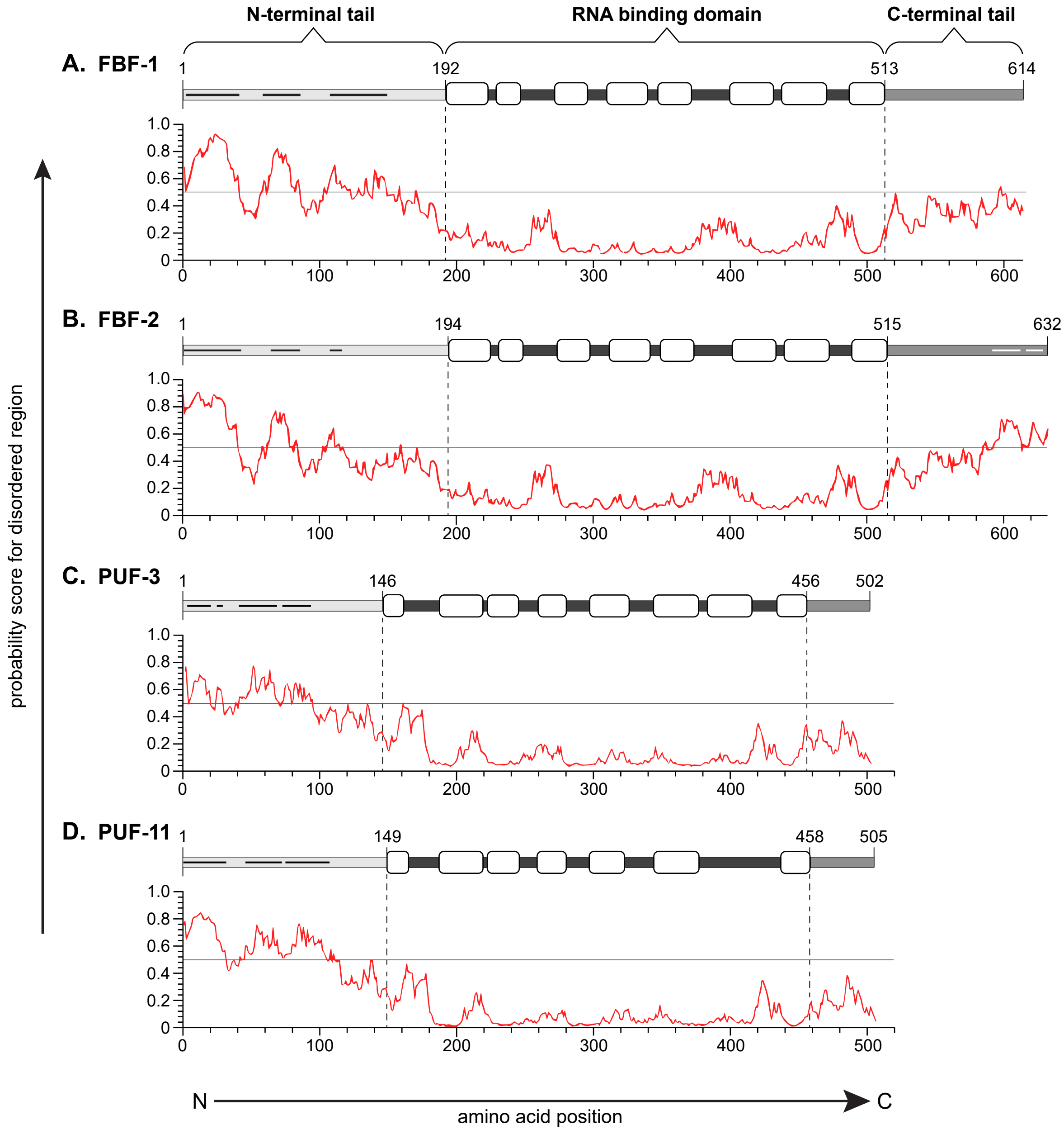

Fig. S6. Predicted intrinsically disordered regions in *C. elegans* PUF proteins specialized for self-renewal.

A-D: Top of each panel, schematic of PUF protein according to PFAM database, with its N-terminal tail (light grey), RNA binding domain with eight PUF repeats (rounded rectangles), and C-terminal tail (dark grey). Lines internal to and along the axis of the protein schematic mark intrinsically disordered regions. Bottom of each panel, probability of intrinsic disorder (red line) according to IUpred3 (using long disorder algorithm with default setting, (Erdős et al., 2021). A probability score of ≥0.5 (horizontal grey line) indicates disordered regions in all N-terminal tails and the C-terminal tail of FBF-2; a probability score of ≤0.5 indicates order regions in all RNA binding domains. Red lines were rendered from the IUpred3 original file in illustrator by image trace

Development • Supplementary information

Table S1. Nematode strains[1] used in this study.

Strain	Genotype	Refer to in the text	Reference
**N2**	*Wild type*	*wild-type*	(Brenner, 1974)
**JK5629**	*glp-1(ar202) [G529E] III/ hT2[qIs48](I;III)*	*Stock*	(Pepper et al., 2003)
	glp-1(ar202)	*glp-1(gf ts)*	
**JK5758**	*lst-1(q895) [lst-1::3XFLAG] I*	*LST-1^{FLAG}*	(Haupt et al., 2019)
**JK5810**	*fbf-2(q945) [3xFLAG fbf-2] II*	*FBF-2^{FLAG}*	*This work*
**JK5929**	*lst-1(q1004) [lst-1::3xV5] I*	*LST-1^{V5}*	(Shin et al., 2017)
**JK5935**	*fbf-1(ok91) [fbf-1 deletion] fbf-2(q945) [3xFLAG fbf-2] II*	*FBF-1 Ø FBF-2^{FLAG}*	*This work*
**JK6002**	*sygl-1(q1015) [sygl-1::1XV5] I*	*SYGL-1^{V5}*	(Shin et al., 2017)
**JK6004**	*sygl-1(q1017) [sygl-1::1xV5::GGS linker::λN] I*	*SYGL-1$^{V5\text{-}\lambda N22}$*	*This work*
**JK6026**	*lst-1(q1028) [λN::lst-1::3xV5] I*	*LST-1$^{V5\text{-}\lambda N22}$*	*This work*
JK6063	*puf-8(q1048) [3xV5::puf-8] II*	*PUF-8^{V5}*	*This work*
**JK6203**	*lst-1(q1125) [(L35A, K80A, L83A)::3xV5] I*	*LST-1 (A^mB^m)^{V5}*	(Haupt et al., 2019)
**JK6270**	*puf-11(q1128) [puf-11::3xV5] IV*	*PUF-11^{V5}*	(Haupt et al., 2020)
**JK6319**	*lst-1(q1004) [LST-1::3xV5] sygl-1(q828) [sygl-1 deletion] I*	*LST-1^{V5} sygl-1(ø)*	(Shin et al., 2017)
**JK6332**	*lst-1(q1004) I; glp-1(ar202) III*	*LST-1^{V5}; glp-1(gf ts)*	*This work*
**JK6367**	*qSi375[(mex-5 promoter::eGFP::linker::his-*	*tethering GFP reporter*	(Aoki et al., 2021)

Development • Supplementary information

	*58::3xboxb::tbb-2 3'utr) *weSi2] II*		
**JK6371**	*sygl-1(q1188): [(I41A, L42A, L79A, L80A) sygl-1::1xV5]*	*SYGL-1(A^{m}B^{m}) ^{V5}*	*This work*
**JK6372**	*lst-1(q1125) I; fbf-2(q945) II; glp-1(ar202) III*	*LST-1 (A^{m}B^{m}) ^{V5}; FBF-2^{FLAG}; glp-1(gf ts)*	*This work*
**JK6373**	*fbf-2(q945) II; glp-1(ar202) III*	*FBF-2^{FLAG} glp-1(gf ts)*	*This work*
**JK6374**	*lst-1(q1004) I; fbf-2(q945) II; glp-1(ar202) III*	*LST-1^{V5}; FBF-2^{FLAG}; glp-1(gf ts)*	*This work*
**JK6385**	*sygl-1(q1162) [(I41A, L42A) sygl-1::1xV5] I*	*SYGL-1 (A^{m}) ^{V5}*	*This work*
**JK6386**	*sygl-1(q1189) [(L79A, L80A) sygl-1::1xV5] I*	*SYGL_1 (B^{m}) ^{V5}*	*This work*
**JK6494**	*lst-1(q895) I; glp-1 (ar202) III*	*LST-1^{FLAG} glp-1(gf ts)*	*This work*
**JK6524**	*ntl-1 (q1236) [ntl-1::3XFLAG] III*	*NTL-1^{FLAG}*	*This work*
**JK6537**	*fbf-1(q1241) [3x FLAG:FBF-1] II*	*FBF-1^{FLAG}*	*This work*
**JK6571**	*fbf-1 (q1248) [3x FLAG:FBF-1] fbf-2 (q738)[deletion of puf repeats]*	*FBF-1^{FLAG} FBF-2 Ø*	*This work*
**JK6579**	*puf-8(q1048) II; glp-1(ar202) III*	*PUF-8^{V5} ; glp-1(gf ts)*	*This work*
**JK6580**	*lst-1(q1004) I; fbf-1(q1241) II; glp-1(ar202) III*	*LST-1^{V5}; FBF-1^{FLAG}; glp-1(gf ts)*	*This work*
**JK6581**	*fbf-1(q1241) II; glp-1(ar202 gf) III*	*FBF-1^{FLAG} ; glp-1(gf ts)*	*This work*
**JK6582**	*lst-1(q895) I; glp-1(ar202) III; puf-11(q1128) IV*	*LST-1^{FLAG}; glp-1(gf ts); PUF-11^{V5}*	*This work*
**JK6583**	*glp-1(ar202) III; puf-11(q1128) IV*	*PUF-11^{V5} glp-1(gf ts)*	*This work*

Development • Supplementary information

**JK6584**	*ntl-1(q1244) [3XFLAG] glp-1(ar202) III*	*NTL-1^{FLAG} glp-1 (gf ts)*	*This work*
**JK6618**	*lst-1(q1265) [lst-1(λN: L35A, K80A, L83A)::3xV5] I*	*LST-1(A^{m}B^{m})$^{V5\text{-}\lambda N22}$*	*This work*
**JK6641**	*lst-1(q1004) I; qSi375II*	*LST-1^{V5}; tethering reporter*	*This work*
**JK6642**	*lst-1(q1028) I; qSi375II*	*LST-1$^{V5\text{-}\lambda N22}$; tethering reporter*	*This work*
**JK6643**	*lst-1(q1125) I; qSi375 II*	*LST-1 (A^{m}B^{m}) ^{V5}; tethering reporter*	*This work*
**JK6644**	*lst-1(q1265) I; qSi375 II*	*LST-1(A^{m}B^{m})$^{V5\text{-}\lambda N22}$; tethering reporter*	*This work*
**JK6696**	*lst-1(q1004) I; ntl-1(q1244) glp-1(ar202) III*	*LST-1^{V5} ; NTL-1^{FLAG} glp-1 (gf ts)*	*This work*
**JK6697**	*lst-1(q1125) I; ntl-1(q1244) [3XFLAG] glp-1(ar202) III*	*LST-1(A^{m}B^{m}) ^{V5}; NTL-1^{FLAG} glp-1 (gf ts)*	*This work*
**JK6698**	*lst-1(q1004) sygl-1(q828) I; glp-1(ar202) III*	*LST-1^{V5} sygl-1(ø); glp-1 (gf ts)*	*This work*
**JK6700**	*lst-1(q1125) I; fbf-1(q1241) II; glp-1(ar202 gf) III*	*LST-1 (A^{m}B^{m}) ^{V5}; FBF-1^{FLAG}; glp-1(gf ts)*	*This work*

[1]Strains are available upon request.

Development • Supplementary information

Table S2. CRISPR guide RNA and repair oligos used in this work.

gene	strain	guide RNA sequence (5'-3')	DNA repair oligo sequence (5'-3')[1]
puf-8	*q1048*	uugaaaucggacgacucau c	ctaaagtaattattttaggggtaatctgcgttcctgA TGggtaagcctatccctaaccctctcctcggtctA gatAGTacTggAaagccAatcccAaacccAc tcctcggActtgatAGCacCggtaagcctatccc taacccActcctcggActtgatAGCacCTCAA GACCAATTTCAATTGGGAATACATG CACATT
fbf-1	*q1241*	aagcccacatctacagaggt	atgctgcgccatcggcgaatgtgcgggagtatgat gatttccCTTGTCATCGTCATCCTTGT AATCGATGTCATGATCTTTATAATC ACCGTCATGGTCTTTGTAGTCaacct ctgtagatgtgggcttttgcggagtctgtaaaattta
fbf-2	*q945*	gatctttataatcaccgtca	aatattattttcatattcccaatcattctaataaaattat caactaatcgacATGGACTACAAAGACC ATGACGGTGATTATAAAGATCATGA CATCGATTACAAGGATGACGATGA CAAGGATCAATCTAAAATGCGAAG AACAAATCAGTTCAGAAAAgtaagttttc agtaacttctaaagaaacaaatggcctaag
ntl-1	*q1244*	accagatgagaaaaatcagt	aataccggagctgctaatcaacagcaaaaccca aacacAaacGACTACAAAGAtCATGAC GGTGATTATAAAGATCATGACATC GATTACAAGGATGACGATGACAAGt gatttttctcatctggtctttcggttcccgattttctgatc
lst-1	*q1028*, *q1265*	aaaagtgaactcactcttgg **agg**	caaaagaattatttgactattcaatcttcgcgcgag acaATGGGAAACGCCCGTACCCGT CGTCGTGAGCGTCGTGCCGAGAA

Development • Supplementary information

			GCAAGCCCAATGGAAGGCCGCCA ACGGAGGATCCTGTTCGACTTTTA TTCTTCCTCCTCGTgtgagttcactttttttttg aaattaaatatgtattttctct
sygl-1	*q1162*	aatctgcttgcggacgcctc	cgccacgtcatcatcgtcaacaggaggcgtccgc GCCGCCacgttgaagccaaagcaaccgaat ggttatgtgcaacact
sygl-1	*q1188*	caccggaaccgggacaccgc	atcttcggtgatttccggcggtgtcccggttccggtg acTGCCGCCgagttgaagaagaaggatcgt agcgagaagaatgtggtga
sygl-1	*q1189*	caccggaaccgggacaccgc	agtgttgcacataaccattcggttgctttggcttcaac gtGAGAATctgcttgcggacgcctcctgttgacg atgatgacgtggcg
sygl-1	*q1017*	cuacugcaaauaauagcug uguuuuagagcuaugc	gaacaacaacacttcactgatgatgggctcTaac agctattatttgcagggtaagcctatccctaaccctc tcctcggtctagatagtactGGAGGATCCGG AAACGCCCGTACCCGTCGTCGTGA GCGTCGTGCCGAGAAGCAAGCCC AATGGAAGGCCGCCAACtagagcgtac ttgctcttttaaattttctaatcc

[1]Uppercase letters denote mutations (including insertions, PAM mutations and/or seed sequence mutations)

Development • Supplementary information

Table S3. Plasmids used in this work for yeast two-hybrid assay

Plasmid	Insert Description	Cloning Site	Vector Backbone	Reference
**pJK1580**	HA::SYGL-1 (a.a. 1-206)	*Nco* I	pACT2	(Shin et al., 2017)
**pJK1581**	HA::SYGL-1 (a.a. 1-103)	*Nco* I	pACT2	This work
**pJK1582**	HA::SYGL-1 (a.a. 103-206)	*Nco* I	pACT2	This work
**pJK2094**	HA::SYGL-1 (a.a. 1-206), A Site	*Nco* I	pACT2	This work
**pJK2095**	HA::SYGL-1 (a.a. 1-206), B Site	*Nco* I	pACT2	This work
**pJK2096**	HA::SYGL-1 (a.a. 1-206), AB Site	*Nco* I	pACT2	This work
**pJK2046**	V5::FBF-2 (a.a. 121-632)	*Nde I*	pBTMKnDB	(Haupt et al., 2019)

Development • Supplementary information
