# Supplementary material for: The in vivo functional significance of PUF hub partnerships in C. elegans germline stem cells
Source: Development. 2023 May 9;150(9):dev201705. doi: 10.1242/dev.201705 (PMC10259659; doi:10.1242/dev.201705)
Supplement: Supplementary file 1 [file develop-150-201705_review_history.pdf]

The *in vivo* functional significance of PUF partnerships in *C. elegans* germline stem cells

Ahlan S. Ferdous, Stephany J. Costa Dos Santos, Charlotte R. Kanzler, Heaji Shin, Brian H. Carrick, Sarah L. Crittenden, Marvin Wickens and Judith Kimble
DOI: 10.1242/dev.201705

Editor: Swathi Arur

Review timeline

Original submission:	15 February 2023
Editorial decision:	20 March 2023
First revision received:	27 March 2023
Accepted:	29 March 2023

Original submission

First decision letter

MS ID#: DEVELOP/2023/201705

MS TITLE: Functional significance of PUF partnerships in*C. elegans* germline stem cells

AUTHORS: Ahlan S Ferdous, Stephany J Costa Dos Santos, Charlotte R Kanzler, Heaji Shin, Brian H Carrick, Sarah L Crittenden, Marvin Wickens, and Judith Kimble

I have now received all the referees reports on the above manuscript, and have reached a decision. The referees' comments are appended below, or you can access them online: please go to BenchPressand click on the 'Manuscripts with Decisions' queue in the Author Area.

The overall evaluation is positive and the reviewers provide recommendations that will enhance the mansucript. Primarily, as Reviewer 1 states the title is too generic and does not specifically capture the advance specific to the paper. A revised title which specifically captures the in vivo relevance of the work should be provided. In addition, as recommended by Reviewer 2, a table to capture the interactions and display them will be useful, in addition to the additional recommendations by the reviewer. Please attend to all of the reviewers' comments in your revised manuscript and detail them in your point-by-point response. If you do not agree with any of their criticisms or suggestions explain clearly why this is so. If it would be helpful, you are welcome to contact us to discuss your revision in greater detail.

Reviewer 1

Advance summary and potential significance to field

Ferdous et al. characterize molecular interactions involved in the regulatory cascade by which Notch signaling maintains C. elegans germ line stem cells in an undifferentiated state. This study builds upon the work of the Kimble and Wickens labs, who previously showed that Notch targets LST-1 and SYGL-1 physically interact with PUF proteins FBF-1/FBF-2 in yeast two-hybrid assays are required in vivo for FBF target repression, and require FBF for in vivo tumor formation (Shin et al., 2017). The Kimble and Wickens labs also demonstrated that LST-1 contains two "KXXL" motifs that are necessary for binding to FBF-1/FBF-2 and for maintenance of an undifferentiated germline stem

© 2023. Published by The Company of Biologists under the terms of the Creative Commons Attribution License (https://creativecommons.org/licenses/by/4.0/).

cell state (Haupt et al., 2019). Similarly, the Traci Hall lab also characterized FBF-2/LST-1/RNA complexes (Qiu et al., 2019).

In their manuscript, Ferdous et al. confirm the in vivo existence and relevance of LST-1/PUF complexes and provide some mechanistic details about the protein "partners" and domains required for regulatory complex assembly and target mRNA repression. Furthermore, they show that SYGL-1 displays PUF interactions that are very similar to LST-1/PUF interactions. Overall, this work extends our understanding of the regulatory mechanisms maintaining C. elegans germ line stem cells, and the field will certainly find the manuscript useful and of high quality. That being said, the findings are largely confirmatory in nature and their broader significance may not rise to the standard of a Development paper.

Comments for the author

The manuscript by Ferdous et al investigates some of the physical interactions between RNA-regulating proteins in C. elegans germ line stem cells, and their requirement for repression of certain targets and maintenance of the cells in an undifferentiated state.
The study largely consists of immunoprecipitation experiments, performed on full-length proteins expressed in a largely-native context, using wild-type and mutant alleles engineered to abrogate some interactions. The authors confirm that interactions already characterized in yeast and in vitro do apply to worm germ cells. Furthermore, they manipulate these interactions and characterize the effect on gene and germ cell regulation; the results confirm their overall model.
The experiments appear to be of a high standard, and the manuscript is well polished and reads smoothly. My only suggestion for improvement is that it may be helpful for the manuscript to include more explicit mention of some limitations in terms of the interpretation of the findings and their broader significance. Specifically:
- The title of the paper should be more specific; the functional significance of PUF interactions with other proteins in C. elegans germline stem cells is already known, thanks in large part to the Kimble and Wickens labs. The title does not capture the advance specific to the present manuscript.
- The protein-protein interactions studied in this manuscript are likely just the tip of the iceberg in terms of physical interactions necessary for normal function of the germline stem cells. The findings reported in this manuscript are certainly worth publishing, but it is not clear that insights gained extend far beyond the specific proteins that were studied (even if the proteins have homologs in multiple species other than C. elegans), or that there are new principles that emerge from this study that are of broad applicability.
- Because of technical limitations, it was necessary to express proteins of interest above their normal total levels and to use tumorous mutants. This could certainly, at least in theory, disrupt complex stoichiometry and lead to spurious findings.
- The concept of a "PUF hub" feels somewhat nebulous and overstated. Yes PUF proteins have many targets, but relatively few inputs to the PUF nodes have been shown to exist.
- The limited scope of functional assays employed, namely the presence or not of undifferentiated cells, probably accounts for the perceived "redundancy" of the regulatory network.

Minor point:
There is a typo on the y axes of Figures 4E and 4F ("DIstal" should be "Distal")

Reviewer 2

Advance summary and potential significance to field

In this review Ferdous et al. describe their analysis of the interactions between PUF proteins and LST-1 and SYGL-1 in the C. elegans germ line. Importantly, this work focused on analyzing the interactions, and the function of the interactions in the worm, building on previous in vitro work. Using co-immunoprecipitation assays, the authors demonstrate that LST-1 interacts with FBF-1, FBF-1 and PUF-11 but does not interact with PUF-8, which is not thought to be part of the PUF hub that regulates the transition from stem cell proliferation to differentiation.
They further demonstrate that two previously identified LST-1 motifs necessary for interaction with the FBF-1 and FBF-2 proteins, are also involved in vivo for interaction. Utilizing a GFP reporter

© 2023. Published by The Company of Biologists under the terms of the Creative Commons Attribution License (https://creativecommons.org/licenses/by/4.0/).

assay, they also demonstrate that recruitment of LST-1 to an RNA results in GFP repression, and that the PUF binding motifs are necessary for this repression, suggesting that PUF proteins are needed to repress. Finally, they demonstrate that recruitment of SYGL-1 to the reporter RNA results in GFP expression, and that either of two motifs are needed for this repression. Overall, manuscript describes experiments that confirm previously described interactions as being important in vivo, and further our understanding of how PUF proteins interact with other proteins to control gene expression. The manuscript is well written, and the conclusions are supported by the results. My suggestions for changes to the manuscript are all relatively minor.

Comments for the author

1. In Figure 1A. proteins are named FBF-3 and FBF-11; however, these should be changed to PUF-3 and PUF-11 2. On page 5 paragraph 3, the authors state 'When tested with the reporter GFP staining was indistinguishable for untethered LST-1(AmBm)V5-LN22…'. I believe that this untethered version should not have LN22; therefore, the protein should just be LST-1(AmBm)V5.
3. In figure 6D, in order to be consistent with the nomenclature used in the results section of the manuscript and to avoid confusion as to the mutant vs. wt form of the motifs, the SYGL protein names should include an 'm'. For example SYGL-1(AmBm), instead of just SYGL-1(AB)
4. The PUF proteins that function with and are able to bind LST-1 and SYG-1 are FBF-1, FBF-2, PUF-3 and PUF-11. These interactions have been demonstrated through yeast 2-hybrid, co-IP (this work). However, not all pairs of interactions have been demonstrated by both of these or other methods. It would be helpful if the authors could provide a table showing each pair of interactions, and which techniques have been used to detect these interactions.
5. Page 5 second paragraph, the authors state '…at a vanishing low level just above background (proximal gonad) (Figure 4B)'. I don't believe the authors are referring to the 'proximal gonad' here, but rather a region of the distal gonad that is more proximal.
6. Page 5 first paragraph- The authors briefly describe the GFP reporter construct and provide the references. It may be beneficial to state that this is an integrated construct.
7. In Figure 1C, it is interesting that the LST-1 levels appear lower in the very distal end of the glp-1(gf ts) gonad. Is this consistently seen, and if so do the authors want to mention this observation and provide a possible explanation?
8. Page 9 first paragraph- The authors state that '…LST-1 localization to the distal gonad restricts PUF-dependent RNA repression to germline stem cell pool'.
However, FBF-1 expression patter is also restricted to the distal end—therefore it is not clear if which is restricting which. The authors may want to qualify this statement a bit.
9. Page 9, bottom paragraph- The authors discuss how the '…LST-1-PUF complex is critical for repression of target RNAs' in reference to when LST-1 and SYGL-1 are tethered. However, it is not clear if the complex is necessary for repression, or if the PUF proteins could repress on their own if they were tethered to the reporter RNA. For this, PUF tethering experiments would need to be performed. This sentence could be rephrased to take into account this unknown.

First revision

<u>Author response to reviewers' comments</u>

Reviewers comments and detailed responses:

Reviewer 1 Advance Summary and Potential Significance to Field:

Ferdous et al. characterize molecular interactions involved in the regulatory cascade by which Notch signaling maintains C. elegans germ line stem cells in an undifferentiated state. This study builds upon the work of the Kimble and Wickens labs, who previously showed that Notch targets LST-1 and SYGL-1 physically interact with PUF proteins FBF-1/FBF-2 in yeast two-hybrid assays, are required in vivo for FBF target repression, and require FBF for in vivo tumor formation (Shin et al., 2017). The Kimble and Wickens labs also demonstrated that LST-1 contains two "KXXL" motifs that are necessary for binding to FBF-1/FBF-2 and for maintenance of an undifferentiated germline stem cell state (Haupt et al., 2019). Similarly, the Traci Hall lab also characterized FBF-

© 2023. Published by The Company of Biologists under the terms of the Creative Commons Attribution License (https://creativecommons.org/licenses/by/4.0/).

2/LST-1/RNA complexes (Qiu et al., 2019).

In their manuscript, Ferdous et al. confirm the in vivo existence and relevance of LST-1/PUF complexes and provide some mechanistic details about the protein "partners" and domains required for regulatory complex assembly and target mRNA repression. Furthermore, they show that SYGL-1 displays PUF interactions that are very similar to LST-1/PUF interactions. Overall, this work extends our understanding of the regulatory mechanisms maintaining C. elegans germ line stem cells, and the field will certainly find the manuscript useful and of high quality. That being said, the findings are largely confirmatory in nature and their broader significance may not rise to the standard of a Development paper.

Reviewer 1 Comments for the Author:

The manuscript by Ferdous et al investigates some of the physical interactions between RNA-regulating proteins in C. elegans germ line stem cells, and their requirement for repression of certain targets and maintenance of the cells in an undifferentiated state.

The study largely consists of immunoprecipitation experiments, performed on full-length proteins expressed in a largely-native context, using wild-type and mutant alleles engineered to abrogate some interactions. The authors confirm that interactions already characterized in yeast and in vitro do apply to worm germ cells.
Furthermore, they manipulate these interactions and characterize the effect on gene and germ cell regulation; the results confirm their overall model.

The experiments appear to be of a high standard, and the manuscript is well polished and reads smoothly. My only suggestion for improvement is that it may be helpful for the manuscript to include more explicit mention of some limitations in terms of the interpretation of the findings and their broader significance. Specifically:

Comment: The title of the paper should be more specific; the functional significance of PUF interactions with other proteins in C. elegans germline stem cells is already known, thanks in large part to the Kimble and Wickens labs. The title does not capture the advance specific to the present manuscript.

> **Response:** Thank you for this recommendation. We revised the title to emphasize its *in vivo* relevance and also to emphasize its analysis of PUF partnerships in the PUF hub. The Revised title is: The *in vivo* functional significance of PUF hub partnerships in *C. elegans* germline stem cells (line 1)

Comment: The protein-protein interactions studied in this manuscript are likely just the tip of the iceberg in terms of physical interactions necessary for normal function of the germline stem cells.

> **Response:** The focus of this manuscript is the PUF hub -- the essential regulatory core required for the function and regulation of germline stem cells (see below). We agree that this core is the tip of the iceberg, but at the same time, we think that understanding the core is essential to other factors.

Comment: The findings reported in this manuscript are certainly worth publishing, but it is not clear that insights gained extend far beyond the specific proteins that were studied (even if the proteins have homologs in multiple species other than C. elegans), or that there are new principles that emerge from this study that are of broad applicability.

> **Response:** The *in vivo* functional significance of individual PUF proteins is well established, but the *in vivo* functional significance of their partnerships is typically inferred from *in vitro* biochemical analyses or yeast molecular analyses that show binding of protein fragments. This work makes the leap by the *in vivo* manipulation of “PUF interacting motifs” in one PUF partner, LST-1, followed by analysis of the consequences of that partnership abrogation. Analogous analyses have never been done to our knowledge. We strongly suspect that principles gleaned from the *in vivo* analysis of LST-1–PUF partnerships reported in this work will have implications for other PUF partnerships throughout the animal kingdom. Yet that prediction must await the test of time.

© 2023. Published by The Company of Biologists under the terms of the Creative Commons Attribution License (https://creativecommons.org/licenses/by/4.0/).

Comment: Because of technical limitations, it was necessary to express proteins of interest above their normal total levels and to use tumorous mutants. This could certainly, at least in theory, disrupt complex stoichiometry and lead to spurious findings.

> **Response:** The tumorous mutants expand LST-1 with a cellular abundance similar to that where it is normally expressed. We have revised the manuscript to make this clear (line 128 and 133).

Comment: The concept of a "PUF hub" feels somewhat nebulous and overstated. Yes, PUF proteins have many targets, but relatively few inputs to the PUF nodes have been shown to exist.

> **Response:** The PUF hub concept is not at all nebulous in our minds and its importance is without doubt. Many questions remain about regulation and function of PUF hub components and also about other network nodes and how they all work together, but those questions do not undermine the PUF hub concept or its importance. The submitted manuscript must not have made that as clear as it needs to be. To address this flaw, the revised manuscript now includes a diagram of the larger network with its self- renewal and differentiation hubs (new Figure 1A) and accompanying text in the Introduction to place the PUF hub in that larger network (lines 55-59). We shifted the former Fig 1A to Fig 1B, and reformatted Figure 1 to accommodate the new Fig 1A, by moving the former Fig 1B to Fig 1E. The revised manuscript also includes a new legend to accompany the new Figure 1A.

Comment: The limited scope of functional assays employed, namely the presence or not of undifferentiated cells, probably accounts for the perceived "redundancy" of the regulatory network.

> **Response:** Individual components of the PUF hub make distinct contributions to when and how many stem cells are maintained. However, they are "redundant with each other" in their abilities to maintain stem cells or not (lines 73-78). Given the tangential nature of their individual contributions for this particular manuscript, we prefer to make no revisions in response to this comment.

Minor point: There is a typo on the y axes of Figures 4E and 4F ("DIstal" should be "Distal")

> **Response:** Many thanks for spotting this. We have revised Figure 4E and 4F as suggested.

Reviewer 2 Advance Summary and Potential Significance to Field:

In this review Ferdous et al. describe their analysis of the interactions between PUF proteins and LST-1 and SYGL-1 in the C. elegans germ line. Importantly, this work focused on analyzing the interactions, and the function of the interactions, in the worm, building on previous in vitro work. Using co- immunoprecipitation assays, the authors demonstrate that LST-1 interacts with FBF-1, FBF-1 2 and PUF- 11, but does not interact with PUF-8, which is not thought to be part of the PUF hub that regulates the transition from stem cell proliferation to differentiation. They further demonstrate that two previously identified LST-1 motifs necessary for interaction with the FBF-1 and FBF-2 proteins, are also involved in vivo for interaction. Utilizing a GFP reporter assay, they also demonstrate that recruitment of LST-1 to an RNA results in GFP repression, and that the PUF binding motifs are necessary for this repression, suggesting that PUF proteins are needed to repress. Finally, they demonstrate that recruitment of SYGL-1 to the reporter RNA results in GFP expression, and that either of two motifs are needed for this repression. Overall, manuscript describes experiments that confirm previously described interactions as being important in vivo, and further our understanding of how PUF proteins interact with other proteins to control gene expression.
The manuscript is well written, and the conclusions are supported by the results. My suggestions for changes to the manuscript are all relatively minor.

Reviewer 2 Comments for the Author:

1. In Figure 1A. proteins are named FBF-3 and FBF-11; however, these should be changed to PUF-3 and PUF- 11

> **Response:** Many thanks for spotting this. Revised Figure 1 corrects this error.

© 2023. Published by The Company of Biologists under the terms of the Creative Commons Attribution License (https://creativecommons.org/licenses/by/4.0/).

2. On page 5 paragraph 3, the authors state 'When tested with the reporter, GFP staining was indistinguishable for untethered LST-1(AmBm)V5-LN22…'. I believe that this untethered version should not have LN22; therefore, the protein should just be LST-1(AmBm)V5.

Response: Many thanks for spotting this. The revised manuscript corrects this error.

3. In figure 6D, in order to be consistent with the nomenclature used in the results section of the manuscript and to avoid confusion as to the mutant vs. wt form of the motifs, the SYGL protein names should include an 'm'. For example, SYGL-1(AmBm), instead of just SYGL-1(AB)

Response: Agreed. The revised Figure 6D corrects this inconsistency.

4. The PUF proteins that function with and are able to bind LST-1 and SYG-1 are FBF-1, FBF-2, PUF-3 and PUF-11. These interactions have been demonstrated through yeast 2-hybrid, co-IP (this work). However, not all pairs of interactions have been demonstrated by both of these or other methods. It would be helpful if the authors could provide a table showing each pair of interactions, and which techniques have been used to detect these interactions.

Response: Thanks for this suggestion. We agree. The revised manuscript includes a table summarizing the prior evidence for the partnerships as Figure S1D, and makes the requisite revisions to text and legends to accommodate the new table.

5. Page 5 second paragraph, the authors state '…at a vanishing low level, just above background (proximal gonad) (Figure 4B)'. I don't believe the authors are referring to the 'proximal gonad' here, but rather a region of the distal gonad that is more proximal.

Response: Now revised to: "more proximal in the gonad", line 198.

6. Page 5 first paragraph- The authors briefly describe the GFP reporter construct and provide the references. It may be beneficial to state that this is an integrated construct.

Response: Now revised to: The reporter RNA, an integrated construct, relies . . .", line 193

7. In Figure 1C, it is interesting that the LST-1 levels appear lower in the very distal end of the glp-1(gf ts) gonad. Is this consistently seen, and if so, do the authors want to mention this observation and provide a possible explanation?

Response: This same distal-most region of *glp-1(gf)* germlines is similarly anomalous for number of mitotic figures (lower than normal) and other proteins (also lower than normal). This effect does not affect our conclusions, and we therefore have made no revisions in response to this comment.

8. Page 9 first paragraph- The authors state that '…LST-1 localization to the distal gonad restricts PUF- dependent RNA repression to germline stem cell pool'. However, the FBF-1 expression pattern is also restricted to the distal end—therefore, it is not clear which is restricting which. The authors may want to qualify this statement a bit.

Response: The FBF and LST-1 patterns are in fact not similarly restricted; both FBF-1 and FBF-2 extend more proximally than LST-1, into the transition zone where germ cells are differentiating and at lower levels even into the pachytene region. We have now added the following sentence on lines 373-374: "For both complexes, distribution of the PUF protein extends well beyond that of its partner."

9. Page 9, bottom paragraph- The authors discuss how the '…LST-1-PUF complex is critical for repression of target RNAs' in reference to when LST-1 and SYGL-1 are tethered. However, it is not clear if the complex is necessary for repression, or if the PUF proteins could repress on their own if they were tethered to the reporter RNA. For this, PUF tethering experiments would need to be performed. This sentence could be rephrased to take into account this unknown.

Response: The point in this section is that LST-1 does not repress RNA on its own in contrast to Nanos, which does repress on its own. To clarify, we expanded the sentence as follows: "but LST-1 does not repress RNA on its own – it needs its PUF partnership (this work)", line 392.

© 2023. Published by The Company of Biologists under the terms of the Creative Commons Attribution License (https://creativecommons.org/licenses/by/4.0/).

Second decision letter

MS ID#: DEVELOP/2023/201705

MS TITLE: The *in vivo* functional significance of PUF partnerships in *C. elegans* germline stem cells

AUTHORS: Ahlan S Ferdous, Stephany J Costa Dos Santos, Charlotte R Kanzler, Heaji Shin, Brian H Carrick, Sarah L Crittenden, Marvin Wickens, and Judith Kimble
ARTICLE TYPE: Research Article

I am happy to tell you that your manuscript has been accepted for publication in Development, pending our standard ethics checks.

© 2023. Published by The Company of Biologists under the terms of the Creative Commons Attribution License (https://creativecommons.org/licenses/by/4.0/).
